# SOX12 and DELTA mediate ecdysone-driven adult neural cell proliferation during metamorphosis

**DOI:** 10.1016/j.jbc.2026.113409

**Published:** 2026-08-07

**Authors:** Lin Wang, Li-Qiang Liang, Pei-Yao Feng, Yan Li, Jin-Xing Wang, Xiao-Fan Zhao

**Affiliations:** Shandong Provincial Key Laboratory of Development and Regeneration, School of Life Sciences, Shandong University, Qingdao, China

**Keywords:** brain, cell proliferation, 20-hydroxyecdyone, SOX12, DELTA

## Abstract

During metamorphic development from larva to adult, insect brains exhibit cell proliferation; however, the molecular mechanisms governing this process remain poorly understood. Employing the lepidopteran insect *Helicoverpa armigera* (cotton bollworm), an agricultural pest, as a model system, this study demonstrates that the steroid hormone 20-hydroxyecdysone (20E) regulates neural cell proliferation during imaginal brain development by upregulating the expression of SOX12 and DELTA *via* nuclear receptor EcRA. SOX12 exhibits significantly elevated expression and is localized in the brain during metamorphosis. Overexpression of SOX12-GFP in the *H. armigera* epidermal cell line (HaEpi) promotes cell proliferation. RNA interference (RNAi)-mediated knockdown of *Sox12* in larvae leads to lethality, delayed pupation, impaired imaginal brain development, downregulation of cell proliferation-related genes, and reduced neural cell proliferation in the brain. CRISPR/Cas9 knockout of *Sox12* can also impair imaginal brain development and reduce neural cell proliferation in the brain. *Sox12* is upregulated by 20E through its nuclear receptor EcRA, which subsequently induces *Delta* expression to facilitate imaginal brain development during metamorphosis. Notably, *Delta* knockdown recapitulates the phenotypic effects observed on *Sox12* silencing and knockout. Collectively, these findings establish that the steroid hormone 20E, acting through the EcRA-SOX12-DELTA regulatory axis, drives neural cell proliferation in the developing imaginal brain during insect metamorphosis.

The precise regulation of postembryonic brain development is the core factor ensuring the construction of nervous system structure, functional maturation, and adaptation to the environment. The postembryonic development of animal brains is a multilevel regulatory process involving gene expression and various regulatory factors, including various hormones. The thyroid hormone is essential for brain development in mammals (1), which promotes fetal neurogenesis (2). Steroid hormones also regulate brain development (3). However, the roles of the steroid hormones in neural cell fate are various. 17β-Estradiol and testosterone promoted hippocampal neural proliferation and improved cell viability *in vitro* (4). Estrogens modulate experimentally induced apoptosis of granule cells in the adult hippocampus (5). Androgens may enhance the survival of newly generated neurons, but not in cell proliferation (6). Determining the roles and demonstrating the mechanism by which steroid hormones regulate postembryonic brain development remains a great challenge.

The insect brain exhibits substantial growth during metamorphic development, transitioning from a small larval brain to a considerably larger adult brain. This growth indicates active proliferation of neural cells, including both neurons and glial cells. Indeed, in *Drosophila*, the adult brain contains approximately 100,000 neurons—an increase of two orders of magnitude compared to the larval brain (7, 8), confirming that neuronal proliferation occurs during the larval-to-adult metamorphosis. 20-hydroxyecdysone (20E) is a steroid hormone that promotes insect metamorphosis. 20E not only promotes larval cell apoptosis, but also promotes adult cell proliferation during metamorphosis. 20E also promotes insect brain remodeling during metamorphosis, including apoptosis and cell proliferation (9); however, the regulatory mechanism of 20E governing neural cell proliferation of the adult brain in the metamorphic development remains incompletely understood.

Many genes are known to be involved in insect adult brain development. For example, sex-determining region Y (SRY)-related high-mobility group (HMG) box (SOX) transcription factors play pivotal roles in regulating cell proliferation, differentiation, and the establishment of stem and progenitor cell populations (10). The SOX family members are functionally involved in both maintaining the multipotency of neural progenitors and promoting neuronal differentiation during embryonic development (11). Furthermore, SOXs appear to have overlapping expression patterns within the developing and adult nervous system, encompassing diverse cell types from the neural stem cell (NSC) stage through to the terminal maturation of neurons and macroglia in mammals (12). SOXs are also implicated in regulation of glial differentiation regulation by facilitating the differentiation of astrocytes and oligodendrocytes (13). Nevertheless, upstream regulatory factors, particularly steroid hormones, and their downstream cascades remain largely uncharacterized in postembryonic brain development.

The 20E-regulated metamorphic brain development presents a good model for studying steroid hormone regulation of postembryonic brain development. In this study, we demonstrate that 20E upregulates *Sox12* expression *via* its nuclear receptor EcRA. Subsequently, SOX12 modulates *Delta* transcription to promote neural cell proliferation. This study revealed a 20E-EcRA-SOX12-DELTA regulatory axis that drives neural cell proliferation to facilitate insect imaginal/adult brain development. This study first clarifies the molecular mechanism by which the steroid hormone upregulates *Sox12* expression *via* the nuclear receptor EcRA, thereby activating Delta signaling to regulate brain development and neural cell proliferation in the adult brain development of the lepidopteran insect *Helicoverpa armigera*, the most serious agricultural insect. It filled the research gap in the molecular mechanisms of neural cell proliferation in postembryonic brain development during insect metamorphosis.

## Results

### Screening of SOX, which was highly expressed in the brain during metamorphosis

Through BLAST (Basic Local Alignment Search Tool) analysis guided by human SOX family members, followed by phylogenetic analysis, nine SOX genes were identified in the *H. armigera* genome (https://blast.ncbi.nlm.nih.gov/Blast.cgi) (Fig. S1). The mRNA expression profiles of these nine SOX genes were subsequently examined in the epidermis, midgut, fat body, and brain tissues *via* quantitative real-time polymerase chain reaction (qRT-PCR) using gene-specific primers (Table S1) to identify SOX family members exhibiting high expression in the brain during metamorphosis. The results revealed that *Sox12* exhibited elevated expression in the sixth instar 96 h (sixth-96 h) across all four tissues, with the most pronounced expression in the brain (Fig. S2 and Table S2). Consequently, SOX12 was selected as the primary focus for further investigation in this study.

### SOX12 was highly expressed in the brain during metamorphosis

**Figure 1:**
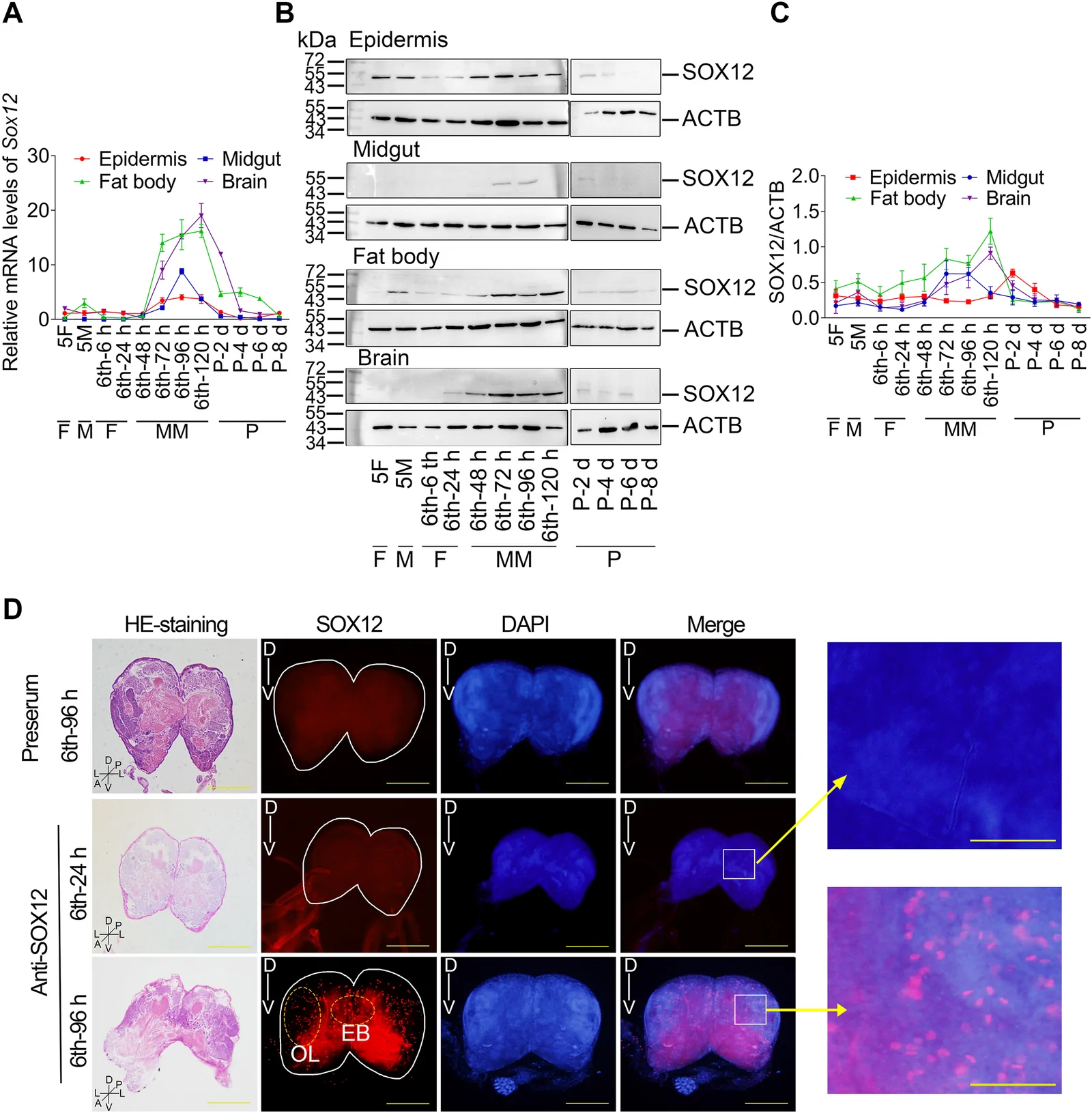
***Sox12* is highly expressed during metamorphosis and localizes in the brain at sixth instar-96 h.***A*, the mRNA levels of *Sox12* in the epidermis, midgut, fat body, and brain during development were detected by qRT-PCR. The bar represents the mean value derived from three independent qRT-PCR replicates, each performed using total RNA extracted from three distinct biological samples. The error bar indicates the mean ± S.D. 5F: fifth instar feeding larvae; 5M: fifth instar molting larvae; sixth-6 h to sixth-120 h: sixth instar 6 h larvae to sixth instar 120 h larvae; P-2 d to P-8 d: pupal stage at day 2 to day 8; F: feeding stage; M: molting stage; MM: metamorphic molting stage; P: pupae. *B*, the protein levels of SOX12 in the epidermis, midgut, fat body, and brain were detected by primary Anti-SOX12 and Anti-ACTB antibodies and goat anti-rabbit IgG HRP conjugate secondary antibodies by western blotting after 12.5% SDS-PAGE. ACTB was used as a protein loading control. *C*, quantification of the data in *B*. The data were quantified based on three independent biological replicates using ImageJ software. *D*, the location of SOX12 detected with immunohistochemistry. Hematoxylin and eosin (HE) staining showing the morphology of the brain. The *white line* represents the shape of the brain, and the *yellow dotted line* represents the optic lobe (OL) and ellipsoid body (EB). Fluorescence was detected using an Olympus BX51 fluorescence microscope (Olympus Optical Co). *Red*, SOX12 detected using anti-SOX12 antibodies and Alexa Fluor 594-Goat Anti-Rabbit IgG; *blue*, nuclei stained using DAPI. The bars represent 200 μm. Back view of the brain. Directions: A, anterior; D, dorsal; L, lateral; P, posterior; V, ventral; ACTB, *β-actin*; DAPI, 4,6-diamidino-2-phenylindole; HRP, horseradish peroxidase; IgG, immunoglobulin G.

To investigate the functional role of SOX12, we initially analyzed its tissue-specific expression patterns during metamorphosis. qRT-PCR analysis revealed that *Sox12* exhibited significantly elevated expression levels in both the brain and fat body, spanning from the 72-h stage of the sixth instar larvae (sixth-72 h, wandering stage) to the 2-day pupae (P-2 d) (Fig. 1*A*). This expression profile was further validated by western blotting, which demonstrated that SOX12 was expressed across multiple tissues, with the highest expression levels observed in the brain from the 72-h to the 120-h stages of the sixth instar larvae (sixth-72 h to sixth-120 h) during metamorphosis (Fig. 1, *B* and *C*). These analyses employed rabbit polyclonal antibodies specifically generated against the *H. armigera* SOX12 protein fragment, as described in this study (Fig. S3). Notably, the experimentally determined molecular weight of SOX12 (50 kDa) was higher than its theoretical prediction (31 kDa), although the underlying reason for this discrepancy remains unclear. The antibody specificity was confirmed through overexpression of SOX12 in HaEpi cells, where the transfected protein was successfully detected by the antibodies (Fig. S4). Collectively, these findings suggest that SOX12 plays a functional role in the metamorphic process.

Immunohistochemical analysis revealed robust SOX12 localization in the brain during the sixth-96 h metamorphic stage, whereas minimal expression was observed in the sixth-24 h brain during the feeding stage (Fig. 1*D*). Consistently, SOX12 exhibited negligible detection in the sixth-24 h larval midgut at the feeding stage, but was abundantly expressed in the imaginal midgut during the sixth-96 h metamorphic period (Fig. S5). These findings suggest that SOX12 plays a significant functional role in imaginal tissue development during metamorphosis.

### Overexpression of SOX12 promoted cell proliferation in HaEpi cells

**Figure 2:**
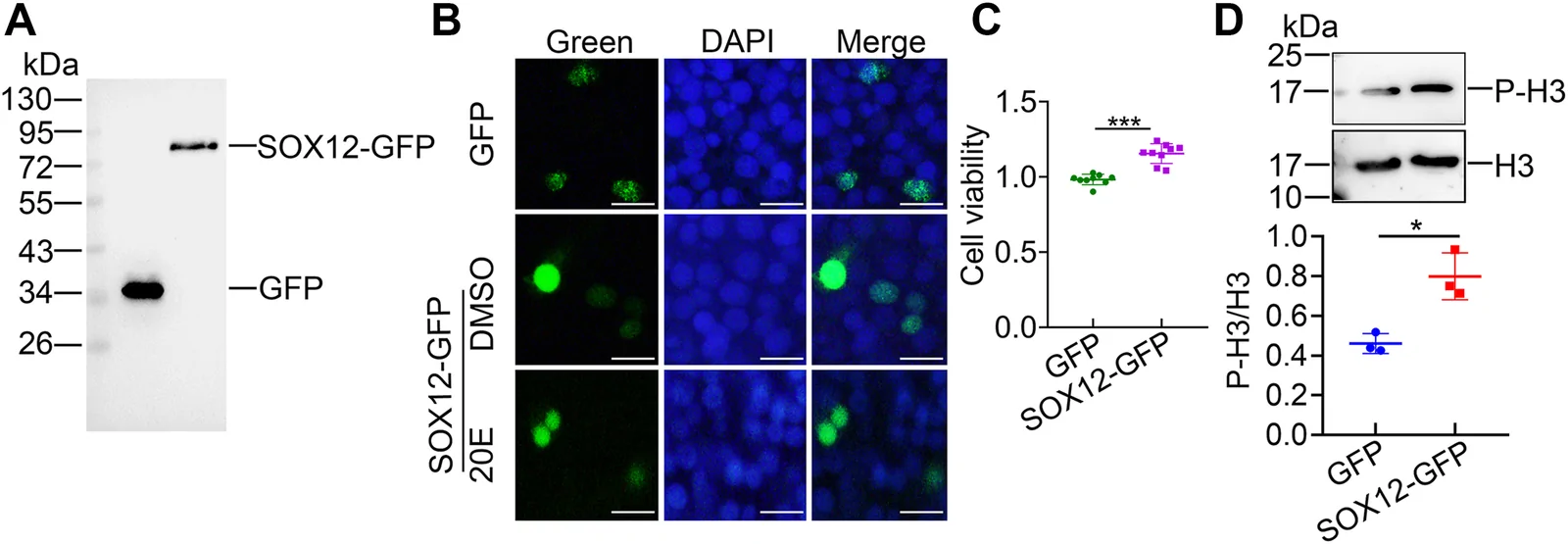
**Overexpression of SOX12-GFP in HaEpi cells promotes cell proliferation.***A*, western blot analysis showing GFP and SOX12-GFP expression in HaEpi cells 72 h after transfection. *B*, subcellular localization of SOX12-GFP after 72 h of overexpression. Fluorescence was detected using an Olympus BX51 fluorescence microscope (Olympus Optical Co). *Green*, SOX12-GFP; *blue*, nucleus stained with DAPI. The bar represents 20 μm. *C*, CCK8 analysis of the cell viability of GFP and SOX12-GFP in HaEpi cells 72 h after transfection. *D*, western blotting showing the band of P-Histone H3 (P-H3) after overexpression of GFP and SOX12-GFP. The statistical analysis was performed using Student’s *t* test (∗, *p* < 0.05, ∗∗, *p* < 0.01, ∗∗∗, *p* < 0.001, n = 3). The error bar indicates the mean ± S.D. CCK8, Cell Counting Kit-8; DAPI, 4,6-diamidino-2-phenylindole.

To investigate the role of SOX12 in cell fate determination, we generated a GFP-tagged SOX12 fusion construct and expressed it in the *H. armigera* epidermal cell line (HaEpi), an established cell line derived from the epidermis of fifth-instar larvae in our laboratory (14) (Fig. 2*A*). Immunofluorescence analysis revealed that SOX12-GFP was predominantly localized to the nucleus, and this subcellular localization remained unaffected by 20E treatment (Fig. 2*B*). Cell viability assays using the Cell Counting Kit-8 (CCK8) demonstrated a significant increase in cell proliferation following SOX12 overexpression (Fig. 2*C*). Furthermore, western blot analysis indicated that SOX12 promoted cell proliferation, as evidenced by elevated levels of phospho-histone H3 (P-H3), a well-established marker of mitotic activity (Fig. 2*D*). Collectively, these findings establish SOX12 as a positive regulator of cell proliferation.

### Knockdown or knockout of *Sox12* impaired neural cell proliferation

**Figure 3:**
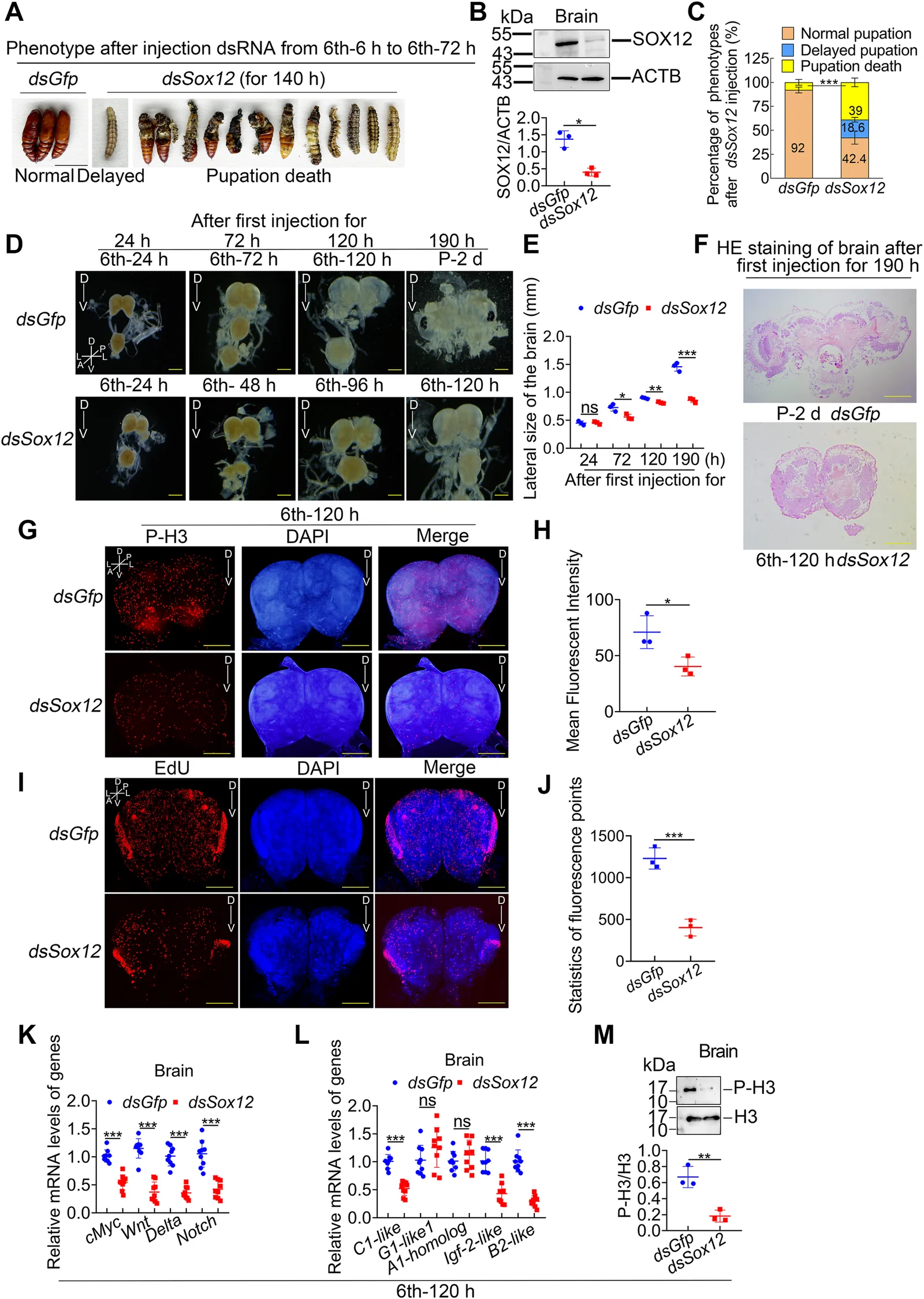
**Knockdown of *Sox12* represses larval-pupal transition and adult brain development.***A*, the phenotypes after *dsGfp* or *dsSox12* injection (sixth-6 h larva to 72 h, 2 μg/larva). The bars represent 1 cm. *B*, efficiency of the knockdown of *Sox12* by western blot analysis. *C*, the statistical analysis of the percentage of different phenotypes after the *dsGfp* or *dsSox12* injection. Each group contained 30 larvae. The data were analyzed using three independent replicates by Student’s *t* test. D, the brain morphology after knockdown of *Sox12*, was observed for 24 h, 72 h, 120 h, and 190 h after the first injection of dsRNA. Sixth-24 h, sixth-48 h, sixth-70 h, sixth-96 h, sixth-120 h, P-2 d indicates the developmental stages of the brain. The bars represent 200 μm. Back view. Directions: A, anterior; D, dorsal; L, lateral; P, posterior; V, ventral. *E*, quantification of the data in (*D*), n = 3. *F*, HE staining of the brain after the first injection for 190 h. Scale bar represents 200 μm. *G*, fluorescence signal of P-H3 in the brain at sixth-120 h by controlling sampling at the same developmental time to avoid the delayed development (120 h after the first injection for *dsGfp* control, and 190 h after the first injection for *dsSox12* injection). Fluorescence was detected using an Olympus BX51 fluorescence microscope (Olympus Optical Co). *Red,* P-Histone H3 (P-H3) detected using anti-P-Histone H3 antibodies and goat anti-rabbit IgG secondary antibody labeled with DyLight594; *blue*, nuclei stained using DAPI. A: anterior, D: dorsal, L: lateral, P: posterior, V: ventral. The scale bar represents 200 μm. *H*, mean fluorescence intensity statistics of *G*, n = 3. *I*, EdU staining DNA replication in the brain at sixth-120 h by sampling at the same developmental time (120 h after the first injection for *dsGfp* control, and 190 h after the first injection for *dsSox12* injection). Fluorescence was detected using an Olympus SpinSR10 laser confocal microscope (Olympus Optical Co). *Red*, EdU staining cells; *blue*, nuclei stained using DAPI. A: anterior, D: dorsal, L: lateral, P: posterior, V: ventral. The scale bar represents 200 μm. *J*, fluorescence points statistics of I, n = 3. *K* and *L*, qRT-PCR shows the expression of cell proliferation-related genes, n = 9 for all graphs (three technical replications and three biological replicates). *M*, western blot and statistical analysis of the expression of P-H3 in the brain. The samples were taken from the brains of sixth-120 h larvae in *dsGfp* and *dsSox12* by controlling sampling at the same developmental time (120 h after the first injection for *dsGfp* control, and 190 h after the first injection for *dsSox12* injection) in *K*-*M*. The statistical analysis was performed using Student’s *t* test (∗, *p* < 0.05, ∗∗, *p* < 0.01, ∗∗∗, *p* < 0.001, n = 3). DAPI, 4,6-diamidino-2-phenylindole; IgG, immunoglobulin G; qRT-PCR, quantitative real-time polymerase chain reaction.

To investigate the role of SOX12 in the brain during metamorphosis, RNA interference (RNAi) was employed by injecting *Sox12* double-stranded RNA (dsRNA) into sixth-instar larvae at 6 h post molt. The primary phenotypic outcome was larval mortality, with a 39% pupal death rate observed. In addition, 18.6% of the treated larvae exhibited a 22-h to 3 days delay in pupation (Fig. 3, *A*–*C*). qRT-PCR analysis confirmed successful *Sox12* knockdown without detectable off-target effects on other *Sox* family members (Fig. S6, *A* and *B*). Morphological analysis revealed significant repression of brain development following *Sox12* knockdown, as evidenced by a notably reduced brain size compared to control specimens (Fig. 3, *D* and *E*). Hematoxylin and eosin (HE) staining showed that brains from knockdown larvae maintained a larval morphology even at 190 h post treatment. In contrast, control brains had progressed to the P-2 d developmental stage (Fig. 3*F*), indicating impaired neural cell proliferation.

To test the hypothesis, cell proliferation in the brain was assessed. Immunohistochemical analysis of phosphorylated histone H3 (P-H3) in the brains of sixth-120 h larvae (120 h post first injection for the *dsGfp* control group, and 190 h post first injection for the *dsSox12* knockdown group, to control the samples at a similar developmental stage) revealed a significant reduction in P-H3 levels following *Sox12* knockdown compared to the control group (Fig. 3, *G* and *H*), suggesting the cell proliferation was repressed by *Sox12* knockdown.

To verify the result, we performed EdU incorporation experiments in the brains of sixth-120 h larvae, which confirmed the repression of cell proliferation detected by P-H3. Because EdU detected DNA duplication, which also indicated the repression of endoreplication by *Sox12* knockdown (Fig. 3, *I* and *J*). Concurrently, the expression of cell proliferation-associated genes, including *cMyc, Wnt, Delta*, and *Notch*, was downregulated upon *Sox12* knockdown (Fig. 3*K*). Furthermore, mRNA levels of insulin-like peptide (ILP) or insulin-like growth factor (IGF) genes *C1-like, Igf-2-like,* and *B2-like* were also decreased following *Sox12* knockdown (Fig. 3*L*). Western blot analysis further confirmed a significant reduction in P-H3 protein levels in the *dsSox12* knockdown group compared to the *dsGfp* control group (Fig. 3*M*). These findings collectively demonstrate that SOX12 plays a pivotal role in promoting neural cell proliferation and the expression of associated genes, thereby facilitating adult brain development during metamorphosis.

**Figure 4:**
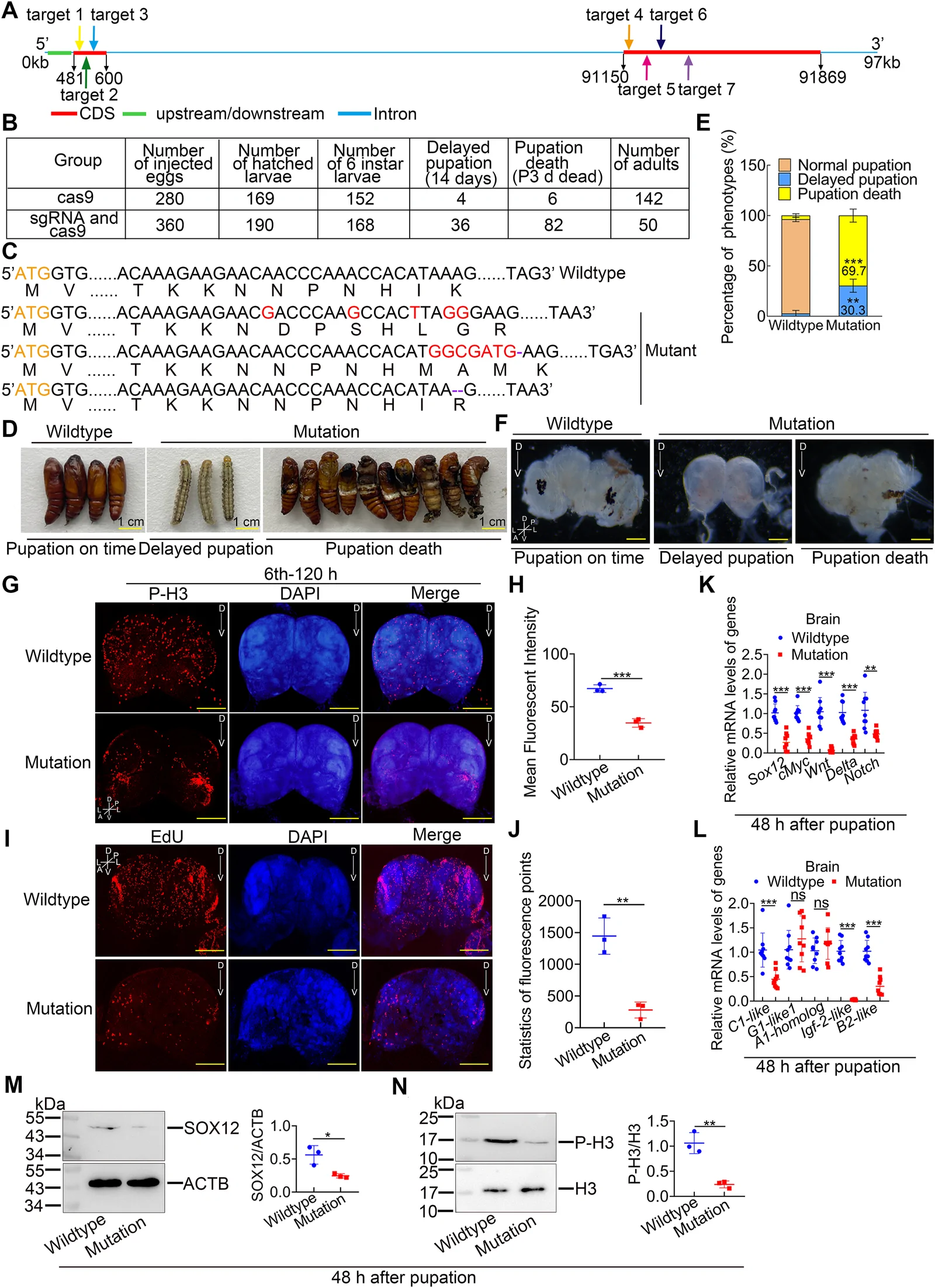
**CRISPR/Cas9-mediated knockout of *Sox12* represses larval-pupal transition and adult brain development.***A*, schematic diagram of the target sites in the exon of the *Sox12* gene. *B*, number of editing and developmental records. *C*, various mutation patterns identified *via* cloning and Sanger sequencing. *Green* represents substitution, *purple* represents insertion. *D*, the phenotype between WT and mutant during the different development times. *E*, the statistical analysis of the percentage of different phenotypes. The data were analyzed using three independent replicates by Student’s *t* test. *F*, the brain morphology between WT and mutant during the different development times. The bars represent 200 μm. Back view. Directions: A, anterior; D, dorsal; L, lateral; P, posterior; V, ventral. *G*, fluorescence signal of P-H3 in the brain of WT in sixth-120 h and mutation in sixth-120 h by sampling at the same developmental time (the mutation was sampled after a 4-day delay). Fluorescence was detected using an Olympus BX51 fluorescence microscope (Olympus Optical Co). The scale bar represents 200 μm. *Red*, P-Histone H3 (P-H3) detected using anti-P-Histone H3 antibodies and goat anti-rabbit IgG secondary antibody labeled with DyLight594; *blue*, nuclei stained using DAPI. A: anterior, D: dorsal, L: lateral, P: posterior, V: ventral. *H*, mean fluorescence intensity statistics of *G*, n = 3. *I,* EdU staining DNA replication in the brain of WT in sixth-120 h and mutation in sixth-120 h by controlling the sampling time (the mutation was sampled after a 4-day delay). Fluorescence was detected using an Olympus SpinSR10 laser confocal microscope (Olympus Optical Co). *Red*, EdU staining cells; *blue,* nuclei stained using DAPI. A: anterior, D: dorsal, L: lateral, P: posterior, V: ventral. The scale bar represents 200 μm. *J*, fluorescence points statistics of *I*, n = 3. *K* and *L*, qRT-PCR shows the expression of cell proliferation-related genes, n = 9 for all graphs (three technical replications and three biological replicates). The samples were taken after pupation for 48 h. *M*, efficiency of the knockout of *Sox12* by western blot analysis. The samples were taken after pupation for 48 h by developmental time control. *N*, western blot and statistical analysis of the expression of P-H3 in the brain. The samples were taken after pupation for 48 h by developmental time control. The statistical analysis was performed using Student’s *t* test (∗, *p* < 0.05, ∗∗, *p* < 0.01, ∗∗∗, *p* < 0.001, n = 3). DAPI, 4,6-diamidino-2-phenylindole; IgG, immunoglobulin G; qRT-PCR, quantitative real-time polymerase chain reaction.

To further confirm that SOX12 plays a role in brain development during metamorphosis, CRISPR/Cas9-mediated knockout was performed by targeting the gene during early embryogenesis. The *Sox12* gene structure comprises two exons and two introns. A total of seven targets were designed in two exon regions (Fig. S7*A*). The sequences of the four targets (target 1, 3, 5, and 7) were successfully cleaved by *in vitro* Cas9 enzyme validation (Fig. S7*B*). Target five was selected for injection by *in vitro* synthesis of single guide RNA (sgRNA) (Fig. 4*A*). Two hundred eighty eggs were injected with Cas9 as control, and 360 eggs were injected with *Sox12*-sgRNA and Cas9 to mutate *Sox12*. In the Cas9 control group, 169 larvae hatched, 152 survived to the sixth instar, four delayed pupations, six died during pupation (pupation death), and 142 successfully became normal adults. However, in the *Sox12*-sgRNA + Cas9 experimental group, 190 larvae hatched and 168 survived to the sixth instar. Thirty-six maintained larval status for 14 days and could not pupate (delayed pupation), and 82 pupation deaths occurred; the editing efficiency was assessed by sequencing everyone using genomic DNA. The other 50 successfully became normal adults, as confirmed by sequencing without editing (Fig. 4*B*). Thymine-Adenine (T-A) clone sequencing identified diverse mutation types, including nucleotide substitutions, insertions, and deletions in *Sox12*, and correlated them with amino acid changes (Figs. 4*C* and S7*D*). PCR amplification of sgRNA-targeted regions revealed clean sequencing peaks in WT specimens, whereas *Sox12* mutants exhibited overlapping peaks at target sites (Fig. S7*C*). The mutation was confirmed by sequencing of the clones produced by PCR, and the reverse primers were designed near the mutation targets. The mutation efficiency in an individual was determined by sequencing five PCR colonies randomly picked from one individual, and a total of 40 individuals were sequenced. The mutation efficiency of the *Sox12* in an individual was 80% (the number of mutated colonies divided by the total number of sequenced colonies × 100% in an individual). The primary phenotypic outcomes were pupation death and delayed pupation (Fig. 4*D*). The editing efficiency in the editing population was 70% (the total number of delayed pupation and pupation death divided by the number of sixth instar larvae × 100%). Among affected larvae (n = 118), 69.7% failed to pupate and died, while 30.3% showed delayed pupation (Fig. 4*E*). Morphological analysis revealed significant repression of brain development following *Sox12* knockout (Fig. 4*F*), suggesting cell proliferation was repressed.

To test the cell proliferation in the brain after knockout of *Sox12*, immunohistochemical analysis of P-H3 in the brains of sixth-120 h larvae by sampling at the same developmental time revealed a significant reduction in P-H3 levels following *Sox12* knockout compared to the Cas9 control group (Fig. 4, *G* and *H*). EdU staining analysis was performed in the brains of sixth-120 h larvae. To exclude the effects of developmental delay, sampling was performed at matched developmental stages. Results revealed that DNA replication was repressed in the *Sox12* knockout sample, compared to the Cas9 control group (Fig. 4, *I* and *J*), suggesting the repression of cell proliferation and DNA duplication by *Sox12* knockout. The expression of cell proliferation-related genes, including *cMyc*, *Wnt*, *Delta*, and *Notch,* was decreased after *Sox12* was confirmed to be knocked out (Fig. 4*K*). In addition, the mRNA level of *C1-like*, *Igf-2-like*, and *B2-like* was decreased after *Sox12* knockout (Fig. 4*L*). Western blot analysis further confirmed a decrease in SOX12 protein expression (Fig. 4*M*) and the P-H3 protein levels (Fig. 4*N*) in edited specimens. These data confirmed the role of SOX12 in neural cell proliferation in the development of the adult/imaginal brain.

### RNA-seq revealed a set of differentially expressed genes in the *Sox12* knockout

**Figure 5:**
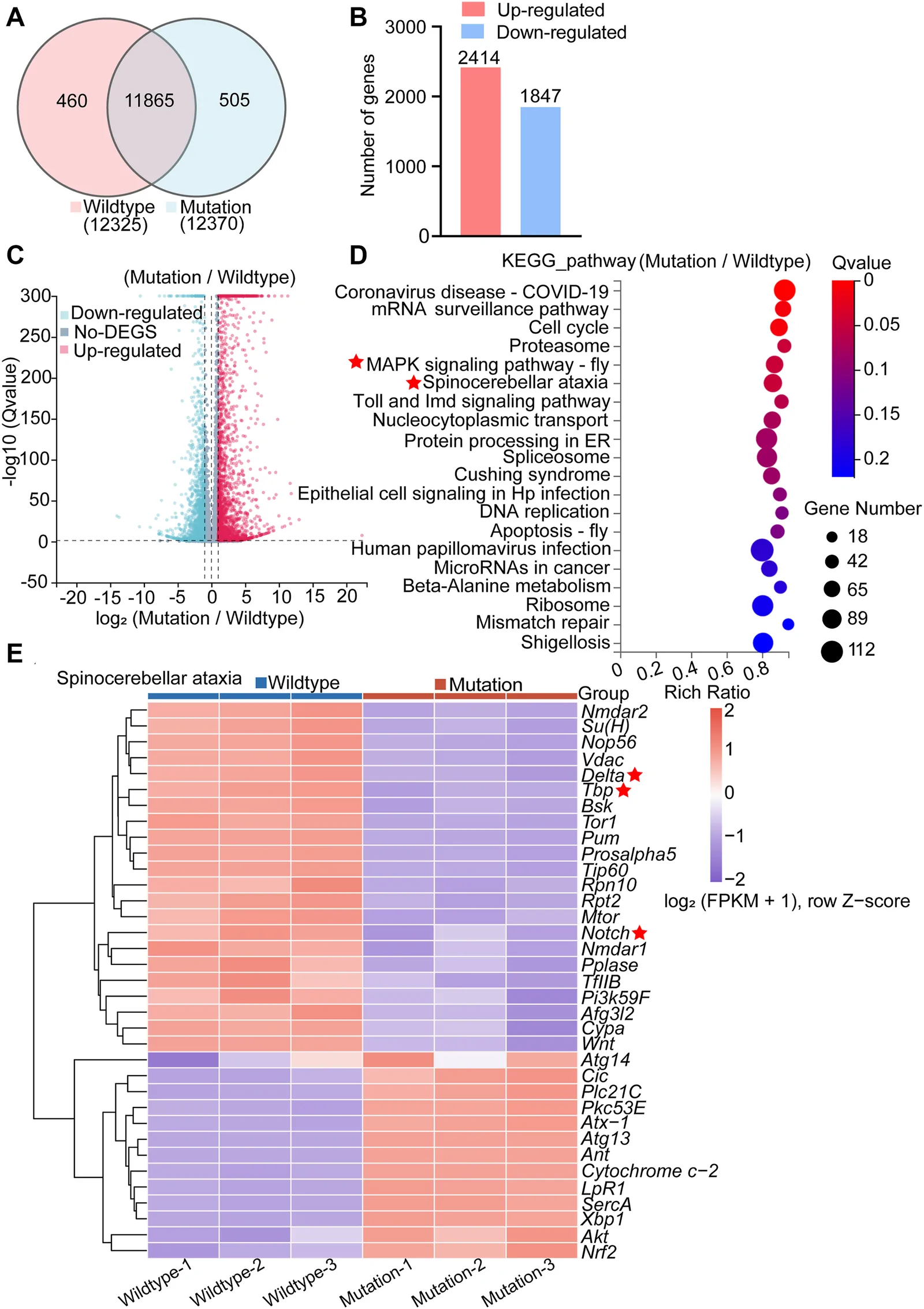
**RNA-seq after CRISPR/Cas9-mediated knockout of *Sox12*.***A*, gene distribution in the WT and mutant by Venn analysis. The genes are listed in Table S4. *B*, number of genes of DEGs, the genes were screened based on log_2_FC (mutation FPKM/wildtype FPKM) ≥1 and Q-value ≤ 0.05. The genes are listed in Table S4. *C*, volcano plots to display the differential genes by identifying the differential genes. The genes are listed in Table S4. *D*, the pathways selected by KEGG based on the Q-value in the data of RNA-seq. The genes are listed in Table S5. *E*, heatmap of genes in the spinocerebellar ataxia pathway, selected by KEGG in the data of RNA-seq. The genes are listed in Table S9. The heatmap was arranged by the log_2_(FPKM+1) value and standardized to Z-scores across samples for each gene. *Red* and *purple* indicate relatively high and low expression, respectively, with the color scale limited to −2 to 2. DEG, differentially expressed gene; FPKM, fragments per kilobase of exon per million reads mapped; KEGG, Kyoto Encyclopedia of Genes and Genomes; RNA-seq, RNA sequencing.

To address the effect of *Sox12* on other gene expression, we analyzed the transcriptome variation after *Sox12* knockout, using Cas9 injection as a normal control. Western blot analysis confirmed a decrease in SOX12 protein expression in edited specimens (Fig. S8*A*). In total, 22.08 million and 22.09 million clean reads were obtained from the brain in WT and mutant, respectively. The clean reads were mapped to the *H. armigera* reference genome, showing that 12,325 genes in the WT and 12,370 genes in the mutant were identified, in which 11,865 genes were commonly shared by WT and mutant, and 460 genes and 505 genes were exclusively expressed in WT and mutant, respectively (Fig. 5*A*, Table S3). Moreover, based on the fragments per kilobase of exon per million reads mapped (FPKM) values of expressed genes, in the common genes, 6002 and 5111 genes were highly expressed in the WT and mutant groups (FPKM≥10), respectively; 3582 and 4169 genes were moderately expressed in the WT and mutant groups (1<FPKM<10), respectively; 3246 and 3550 genes were lowly expressed in the WT and mutant groups (FPKM≤1), respectively (Fig. S8*B*). When the genes with log_2_FC (mutation FPKM/wildtype FPKM) ≥1 and Q-value≤0.05 were defined as differentially expressed genes (DEGs), of the commonly shared genes, 2414 were found to be upregulated genes, and 1847 were downregulated genes in the *Sox12* knockout sample (Fig. 5*B*, Table S4). We created volcano plots to display the differentially expressed genes in the *Sox12* knockout sample (Fig. 5*C*). The Kyoto Encyclopedia of Genes and Genomes (KEGG) metabolic pathways involving the differentially expressed genes were divided into seven branches: Cellular Processes, Environmental Information Processing, Genetic Information Processing, Human Disease (limited to animals), Metabolism, Organismal Systems, and Drug Development. Further classification and statistics were conducted under each branch. The annotation classification results of the KEGG pathways for the DEGs were plotted (Fig. S8*C*, Table S5). Based on the KEGG pathway annotation classification, the DEGs were classified by function. Generally, the functions with Q-value ≤0.05 were regarded as significantly enriched (Fig. 5*D*, Table S6). The mitogen-activated protein kinases (MAPKs) signaling pathway, fly-related to cell proliferation, and spinocerebellar ataxia related to the nervous system were selected for further analysis. The heatmap analysis of the MAPK signaling pathway-fly showed that the genes transducin beta-like ebi (*Ebi*), raf homolog serine/threonine-protein kinase (*Raf*), and dual specificity mitogen-activated protein kinase kinase dSOR1 (*Dsor1*), which promote cell proliferation, were downregulated after *Sox12* knockout (Fig. S9, Table S7). We performed a heatmap analysis of all genes in the spinocerebellar ataxia pathway (Fig. S10, Table S8). The genes neurogenic locus protein delta (*Delta*), neurogenic locus Notch protein (*Notch*), and TATA-box-binding protein (*Tbp*), which promote neural cell proliferation, were downregulated after *Sox12* knockout (Fig. 5*E*, Table S9). These data further confirmed that SOX12 plays a pivotal role in promoting neural cell proliferation.

### 20E through EcRA upregulated *Sox12* expression

**Figure 6:**
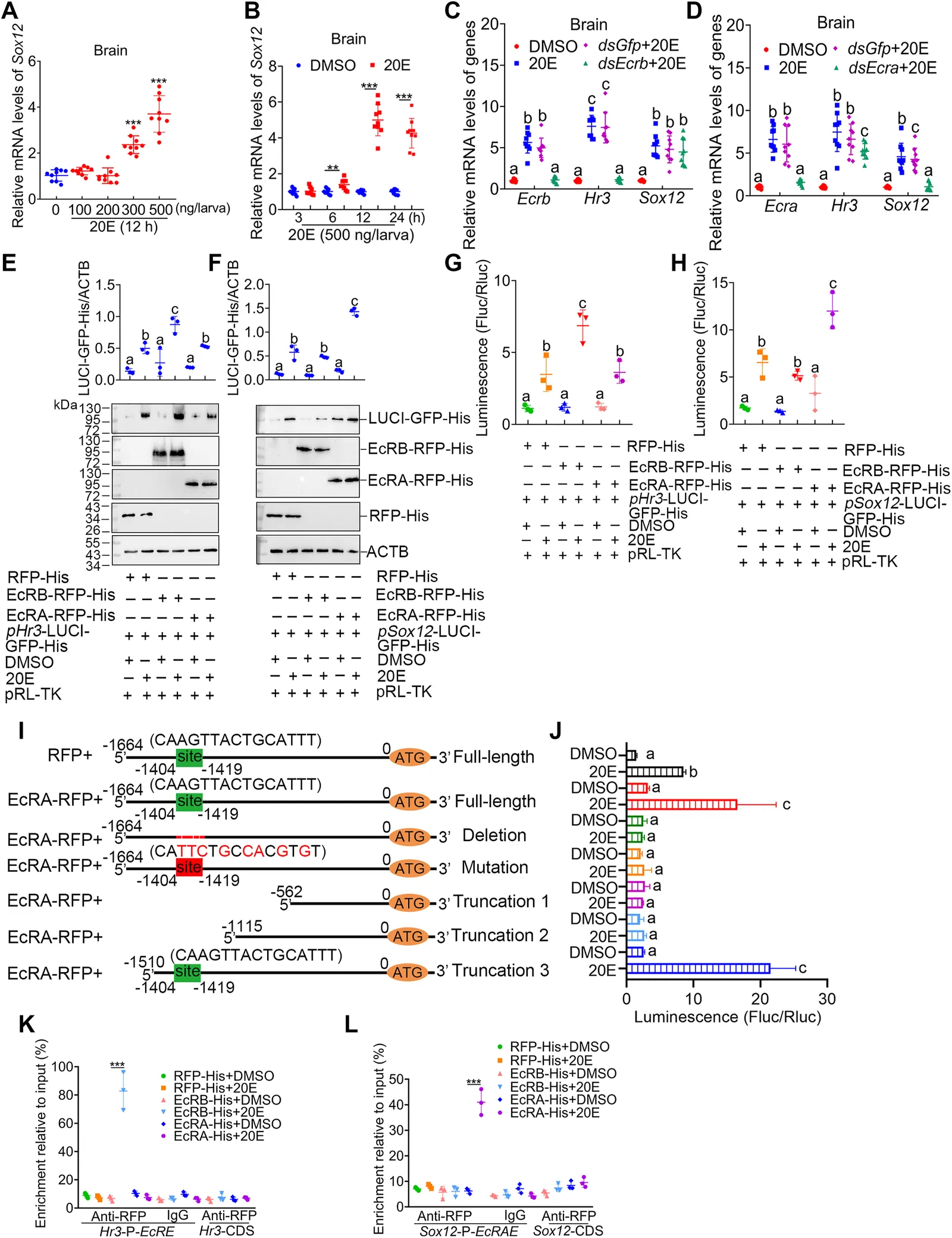
**20E through EcRA upregulates *Sox12* expression.***A*, the expression of *Sox12* in the sixth instar larval brain under stimulation with different concentrations of 20E. *B*, time course of the *Sox12* expression in the sixth instar larval brain after 20E induction. DMSO was used as the solvent control. C, knockdown of *Ecrb* in sixth instar 6 h by *dsEcrb* (3 μg/larva) followed by stimulation with 20E (500 ng/larva) for 12 h to detect the expression of *Hr3* and *Sox12*. *D*, knockdown of *Ecra* sixth instar 6 h by *dsEcra* (3 μg/larva) followed by stimulation with 20E (500 ng/larva) for 12 h to detect the expression of *Hr3* and *Sox12*. *E*, western blotting showed the expression of the *pHr3*-LUCI-GFP-His reporter plasmid (LUCI-GFP-His) under overexpression of EcRB or RFP with 20E or DMSO treatment. *F*, western blotting showed the expression of the *pSox12*-LUCI-GFP-His reporter plasmid (LUCI-GFP-His) under overexpression of EcRA or RFP with 20E or DMSO treatment. *G*, transcriptional activity of the *pHr3*-LUCI-GFP-His reporter plasmid was detected by dual-luciferase reporter assay. The *pHr3*-LUCI-GFP-His vector carries firefly luciferase. The reference pRL-TK vector carries Renilla luciferase. The relative luciferase activity was defined as the Fluc level/Rluc level. Fluc: Firefly luciferase; Rluc: Renilla luciferase. The Rluc was used as an internal reference to eliminate background. *H*, transcriptional activity of the *pSox12*-LUCI-GFP-His reporter plasmid was detected by dual-luciferase reporter assay. The *pSox12*-LUCI-GFP-His vector carries firefly luciferase. The reference pRL-TK vector carries Renilla luciferase. The relative luciferase activity was defined as the Fluc level/Rluc level. Fluc: Firefly luciferase; Rluc: Renilla luciferase. The Rluc was used as an internal reference to eliminate background. *I*-*J*, dual-luciferase reporter assay to measure the transcriptional activity of EcRAE binding sites in different LUCI-GFP-His reporter plasmids. *K*, ChIP assay showed that 20E promotes *Hr3* expression *via* EcRB binding to EcRE. The primers for EcRE are the sequences containing EcRE in the *Hr3* promoter region, respectively. Primer *Hr3* targeting the *Hr3* coding DNA sequence (CDS) was used as a control. *L*, ChIP assay showed that 20E promotes *Sox12* expression *via* EcRA binding to EcRAE. The primers for EcRAE are the sequences containing EcRAE in the *Sox12* promoter region, respectively. Primer *Sox12* targeting the *Sox12* coding DNA sequence (CDS) was used as a control. The statistical analysis was performed using Student’s *t* test (∗, *p* < 0.05, ∗∗, *p* < 0.01, ∗∗∗, *p* < 0.001, n = 3). Statistical analysis of (*C*), (*D*), (*E*), (*F*), (*G*), (*H*), and (*J*) was conducted using two-way Anova from three replicates, with a Bonferroni-corrected *post hoc* test revealed that different characters presented significant differences; different letters represented significant differences (*p* < 0.05). The error bar indicates the mean ± S.D. DMSO, dimethyl sulfoxide; EcRE, EcRB-binding element; EcRAE, EcRA-binding element; ChIP, chromatin immunoprecipitation; pRL-TK, pIEx-4-Renilla luciferase thymidine kinase.

Since *Sox12* exhibited high expression levels during metamorphosis, we hypothesized that 20E might regulate its expression because 20E promotes metamorphosis. To test this hypothesis, we investigated the effects of 20E on *Sox12* expression. Our results demonstrated that larval hemolymph injections of 20E (300 and 500 ng) significantly elevated *Sox12* mRNA levels compared to dimethyl sulfoxide (DMSO)-treated controls (Fig. 6*A*). Notably, treatment with 500 ng of 20E induced a sustained upregulation of *Sox12* expression from 6 to 24 h post-treatment in the brain (Fig. 6*B*), providing clear evidence that 20E positively regulates *Sox12* expression.

The broadly characterized ecdysone receptor B (*Ecrb*), a principal nuclear receptor for 20E, was subsequently knocked down to validate the regulatory role of 20E in upregulating *Sox12* expression. Notably, *Sox12* expression levels remained unaffected following *Ecrb* knockdown when *Hr3*—a well-established downstream effector in the EcRB-mediated signaling pathway (15)—was simultaneously targeted (Fig. 6*C*). In contrast, specific knockdown of *Ecra* resulted in a significant reduction of *Sox12* expression, whereas *Hr3* expression levels were not diminished (Fig. 6*D*). These findings collectively indicate that 20E exerts its stimulatory effect on *Sox12* expression predominantly through the EcRA nuclear receptor.

Phylogenetic analysis classified EcRB and EcRA (Fig. S11), and sequence alignment revealed their divergence primarily at the N-terminal regions (Fig. S12). Both *Ecrb* and *Ecra* exhibited high expression levels in the brain and other tissues during metamorphosis. Notably, under identical qRT-PCR template conditions, *Ecra* demonstrated significantly higher expression than *Ecrb* during this developmental stage (Fig. S13, *A* and *B*). The RNAi efficiency, off-target effects, and 20E-inducibility of *Ecrb* and *Ecra* were systematically assessed through qRT-PCR analyses (Fig. S13, *C*–*H*).

To further investigate the involvement of EcRA in 20E-mediated upregulation of *Sox12*, the promoter sequence of *Sox12* was analyzed using the JASPAR database (http://jaspar.genereg.net/) to identify potential EcR binding sites by targeting the ecdysone response element (EcRE) motif. A putative EcRE was predicted within the promoter region of *Sox12* (5′-1419-CAAGTTACTGCATTT-1404-3′), and it was consistent with the sequencing results, which was designated as EcRAE in this study. Notably, this EcRAE sequence exhibited limited conservation with the canonical EcRE consensus (5′-1401-GGGGTCAATGAACTG-1386-3′) previously reported in the promoter regions of *Drosophila melanogaster* and *H. armigera* (15, 16, 17).

To functionally characterize these elements, luciferase reporter plasmids fused with green fluorescent protein (LUCI-GFP-His) were constructed, incorporating either the 5-upstream sequences of *Hr3* (0-1864) and *Sox 12* (0-1664), with *Hr3* EcRE (*p*Hr3-LUCI-GFP-His) or the predicted *Sox12* EcRAE (*pSox12*-LUCI-GFP-His) (Fig. S14). In addition, overexpression plasmids encoding RFP-tagged EcRA (EcRA-RFP-His) and EcRB (EcRB-RFP-His) were generated and cotransfected into HaEpi cells along with the respective luciferase reporter constructs (*pHr3*-LUCI-GFP-His or *pSox12*-LUCI-GFP-His) to assess the regulatory roles of EcR isoforms (EcRA and EcRB) in 20E-dependent upregulation of Sox12 expression.

Western blot analysis revealed a significant upregulation of luciferase reporter (LUCI-GFP-His) expression in the *pHr3*-LUCI-GFP-His construct (containing the EcRE) upon 20E induction when EcRB-RFP-His was overexpressed, relative to EcRA-RFP-His expression (Fig. 6*E*). This finding suggests that 20E induces reporter expression through EcRB binding to the EcRE. In contrast, reporter (LUCI-GFP-His) expression in the *pSox12*-LUCI-GFP-His construct (containing the EcRAE) was markedly upregulated under 20E induction when EcRA-RFP-His was overexpressed, compared to EcRB-RFP-His expression (Fig. 6*F*). These results indicate that 20E promotes *Sox12* expression *via* EcRA binding to the EcRAE. The luciferase activity assay showed, in the RFP-His tag control group, both *pHr3*-LUCI-GFP-His and *pSox12*-LUCI-GFP-His reporter plasmids exhibited elevated expression levels following 20E induction compared to DMSO treatment (Fig. 6, *G* and *H*), yielding results consistent with those of the Western blot analysis.

To further confirm the luciferase activity of EcRAE, we constructed the luciferase reporter plasmids of deletion, mutation, truncation 1, truncation 2, and truncation 3 (Fig. 6*I*). The transcriptional activity of the full-length plasmid and the truncation 3 plasmid, both of which had EcRAE element, were significantly increased following 20E induction compared to DMSO treatment, whereas other truncations, deletion, or mutation of EcRAE had little transcriptional activity (Fig. 6*J*), suggesting EcRAE was essential for EcRA transcriptional activity. The expression of LUCI-GFP-His in RFP-His expression under 20E induction is due to the endogenous EcRA or EcRB.

Chromatin immunoprecipitation (ChIP) assays further demonstrated that EcRB-RFP-His exhibited enhanced binding to EcRE elements in the *Hr3* promoter under 20E treatment relative to other treatments (Fig. 6*K*), whereas EcRA-RFP-His showed increased binding to EcRAE elements in the *Sox12* promoter following 20E induction compared to alternative treatments (Fig. 6*L*). These findings collectively indicate that 20E-mediated upregulation of *Sox12* transcription occurs through EcRA binding to EcRAE elements.

### SOX12 upregulates *Delta* expression

**Figure 7:**
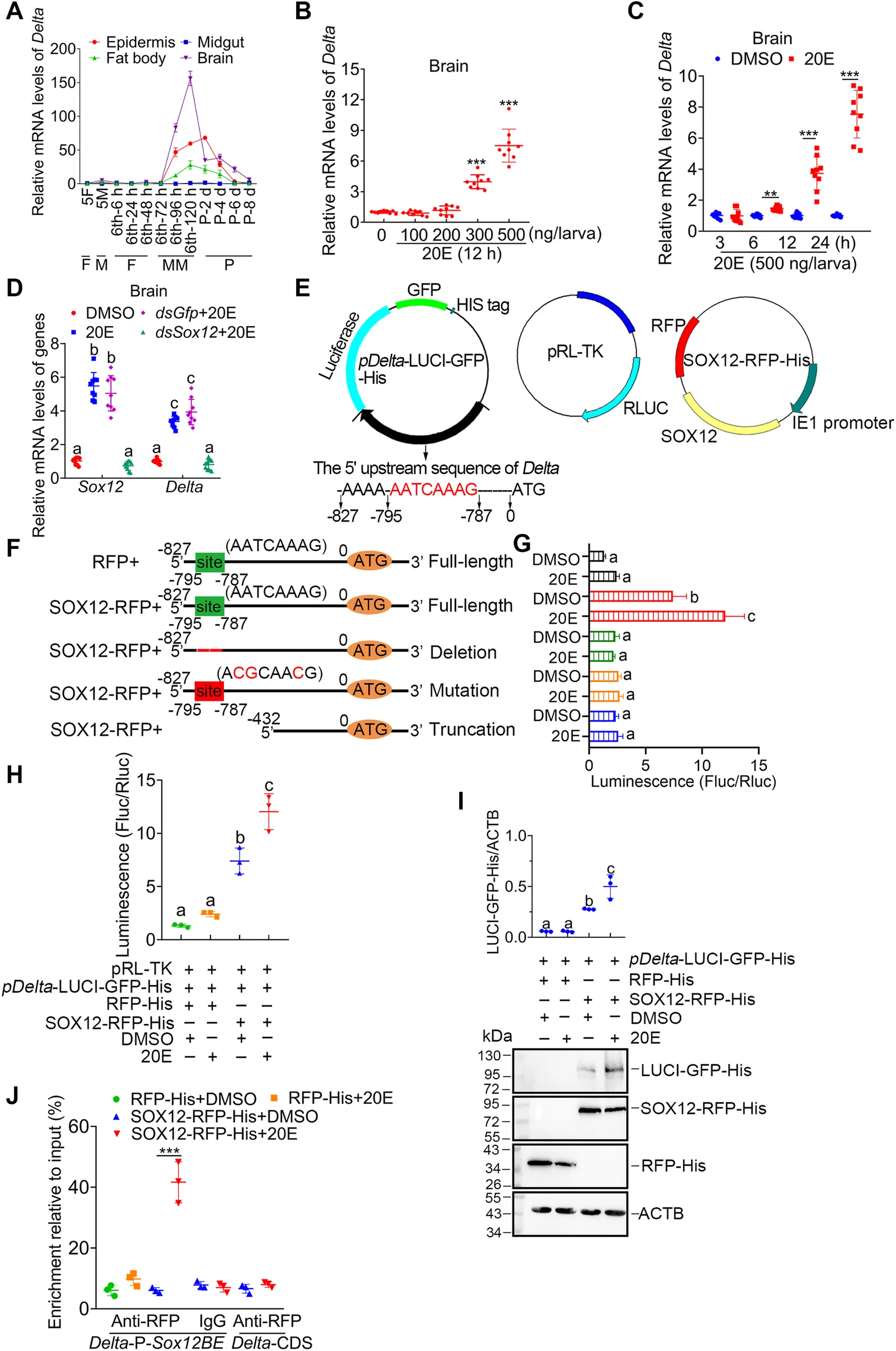
**20E upregulates Delta expression through SOX12.***A*, the expression of *Delta* in the epidermis, midgut, fat body, and brain. 5 F: fifth instar feeding larvae; 5 M: fifth instar molting larvae; sixth instar 6 h larvae to sixth instar 120 h larvae; P-2 d to P-8 d: pupal stage at day 2 to day 8; F: feeding stage; M: molting stage; MM: metamorphic molting stage; P: pupae. The bar represents the mean value derived from three independent qRT-PCR replicates, each performed using total RNA extracted from three distinct biological samples. The error bar indicates the mean ± S.D. *B*, the expression of *Delta* in the brain under stimulation with different concentrations of 20E for 12 h. *C*, time course of the *Delta* expression in the brain after 20E (500 ng/larva) induction. DMSO was used as the control. *D*, knockdown of *Sox12* in the brain by *dsSox12* (3 μg/larva) followed by stimulation with 20E (500 ng/larva) for 12 h to detect the expression of *Delta*. *E*, plasmid maps of *pDelta*-LUCI-GFP-His, SOX12-RFP-His, and pRL-TK. *F*-*G*, dual-luciferase reporter assay to measure the transcriptional activity of SOX12BE binding sites in different LUCI-GFP-His reporter plasmids. *H*, transcriptional activity of the *pSox12*-LUCI-GFP-His reporter plasmid was detected by dual-luciferase reporter assay. The *pSox12*-LUCI-GFP-His vector carries firefly luciferase. The reference pRL-TK vector carries Renilla luciferase. The relative luciferase activity was defined as the Fluc level/Rluc level. Fluc: Firefly luciferase; Rluc: Renilla luciferase. The Rluc was used as an internal reference to eliminate background. *I*, western blotting showed the expression of the *pDelta*-LUCI-GFP-His reporter plasmid (LUCI-GFP-His) under overexpression of SOX12 or RFP with 20E or DMSO treatment. n = 3. *J*, ChIP analysis of SOX12 binding to the *Delta* promoter region. Input: nonimmunoprecipitated chromatin. IgG, nonspecific mouse IgG. The primers for *Sox12* were the sequences containing SOX12BE in the *Delta* promoter region, respectively. Primers *Delta* targeting the *Delta* coding DNA sequence (CDS) were used as a control. n = 3. The statistical analysis was performed using Student’s *t* test (∗, *p* < 0.05, ∗∗, *p* < 0.01, ∗∗∗, *p* < 0.001, n = 3). Statistical analysis of (*D*), (*G*), (*H*), and (*I*) was conducted using two-way ANOVA from three replicates, with a Bonferroni-corrected *post hoc* test, which revealed that different characters presented significant differences; different letters represented significant differences (*p* < 0.05). The error bar indicates the mean ± S.D. DMSO, dimethyl sulfoxide; SOX12BE, SOX12-binding element; RLU, relative light unit; ChIP, chromatin immunoprecipitation; IgG, immunoglobulin G; pRL-TK, pIEx-4-Renilla luciferase thymidine kinase; qRT-PCR, quantitative real-time polymerase chain reaction.

The expression levels of *cMyc, Wnt, Delta, Notch*, and *Igfs* were significantly downregulated following *Sox12* knockdown and knockout in the above study, indicating that these genes function as downstream effectors of SOX12. RNA-seq data analysis also indicates that the expression of *Notch* and *Delta* decreased after the knockout of *Sox12*. Among these, *Delta* was selected for further investigation as a potential downstream target of SOX12 due to its notably high expression in the brain during the 6th to 96 h metamorphic stages (Fig. S15). Expression profiling revealed that *Delta* exhibits robust expression in the brain throughout metamorphosis (Fig. 7*A*). Notably, injection of 20E in sixth instar 6 h larvae resulted in a significant upregulation of *Delta* expression in the brain compared to the DMSO control (Fig. 7, *B* and *C*), suggesting that 20E promotes *Delta* expression. Conversely, *Delta* expression was markedly reduced upon *Sox12* knockdown *via* dsRNA injection in sixth instar larvae (Fig. 7*D*), indicating that 20E regulates *Delta* expression in a SOX12-dependent manner.

To investigate the regulatory mechanism by which 20E induces *Delta* expression through SOX12, we first analyzed the promoter sequence of *Delta* and identified a predicted SOX-binding motif (5ʹ-795-AATCAAAG-787-3ʹ, designated as SOXBE) within its promoter region using the JASPAR database (http://jaspar.genereg.net/). This motif exhibited high sequence similarity to the known binding site of *Drosophila* Sox100B (5ʹ-(A/T) (A/T) CAA(A/T) G-3ʹ) (18, 19, 20). To assess the transcriptional regulation of *Delta* by *Sox12*, we engineered a reporter plasmid (*pDelta*-LUCI-GFP-His) by replacing the pIE promoter in the pIEx-4-luciferase-GFP-His vector with the 5ʹ upstream region containing the SOXBE, which was consistent with the sequencing results (Fig. 7*E*). Meanwhile, we also engineered deletion, mutation, and truncation reporter plasmids (Fig. 7*F*). This reporter construct was co-transfected with a SOX12-overexpressing plasmid (pIEx-4-SOX12-RFP-His) into HaEpi cells to evaluate the regulatory effect of SOX12 on *Delta* transcription. Only the transcriptional activity of the full-length plasmid that contains SOXBE was significantly increased following 20E induction compared to DMSO treatment (Fig. 7*G*). Subsequent luciferase activity assays demonstrated that SOX12 significantly enhanced the transcriptional activity of the luciferase reporter under 20E induction (Fig. 7*H*). Western blot analysis confirmed the expression of the LUCI-GFP-His fusion protein in SOX12-RFP-His-overexpressing cells, and 20E increased the expression, whereas no expression was detected in the RFP-His tag control group (Fig. 7*I*). The transcriptional activity of the SOX12BE-containing promoter with DMSO treatment may be due to the SOX12 expression in the nuclei. Furthermore, ChIP assays revealed that SOX12-RFP-His specifically enriched DNA fragments containing the SOXBE motif (Fig. 7*J*). Collectively, these findings provide compelling evidence that SOX12 upregulates *Delta* expression in response to 20E signaling.

### DELTA promoted the cell proliferation of the adult brain

**Figure 8:**
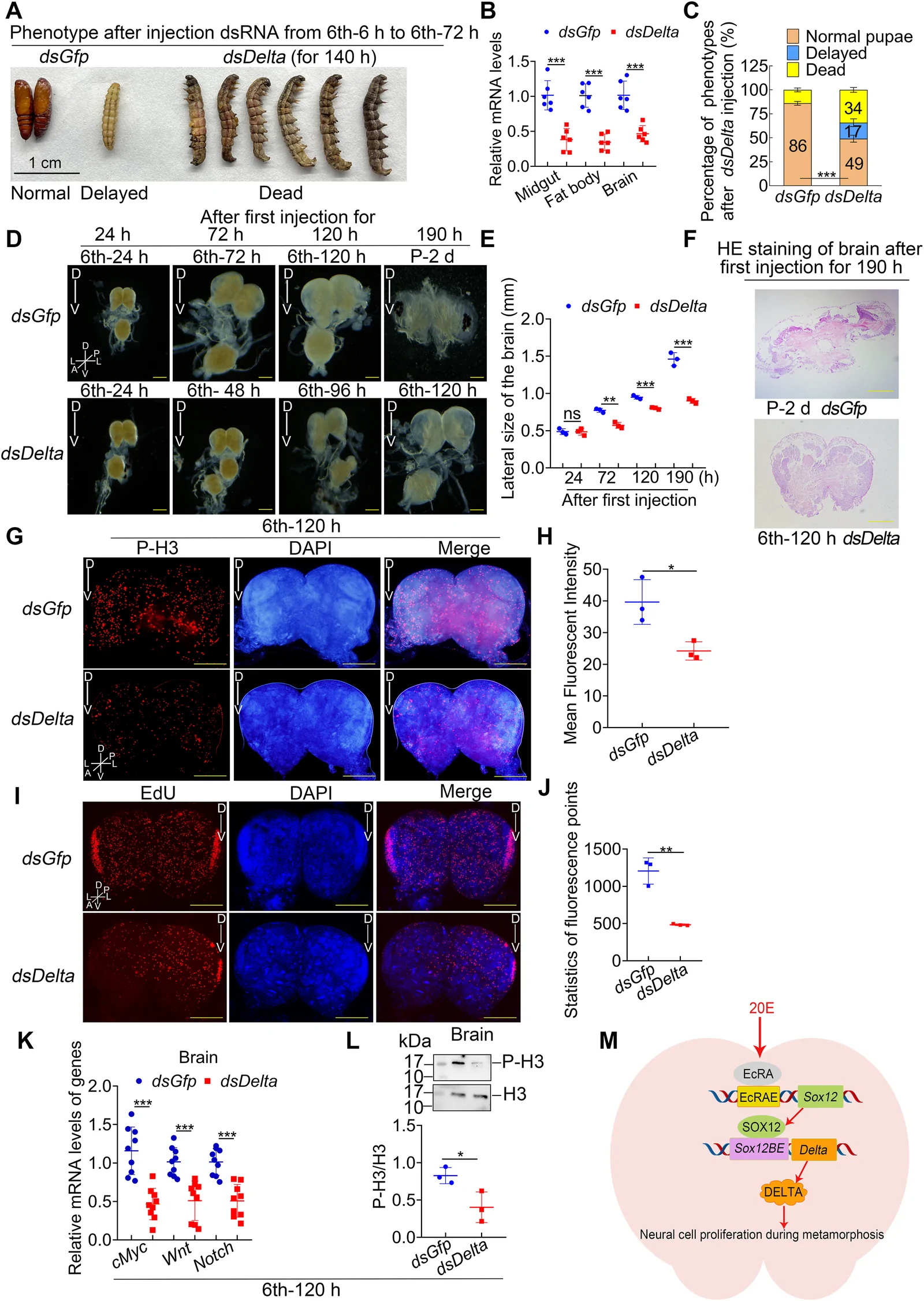
**DELTA promotes the cell proliferation of the adult brain.***A*, the phenotypes after *dsGfp* or *dsDelta* injection from sixth-6 h larva to 72 h (2 μg/larva). *B*, efficiency of the knockdown of *Delta* by qRT-PCR analysis. *C*, the statistical analysis of the percentage of different phenotypes after the *dsGfp* or *dsDelta* injection. Each group contained 30 larvae. The data were analyzed using three independent replicates by Student’s *t* test. *D*, the brain morphology, after knockdown of *Delta* for 24 h, 72 h, 120 h, and 190 h from the first injection of dsRNA. Sixth-24 h, sixth-48 h, sixth-70 h, sixth-96 h, sixth-120 h, and P-2 d indicate the developmental stages of the brain. The bars represent 200 μm. Back view. Directions: A, anterior; D, dorsal; L, lateral; P, posterior; V, ventral. *E*, quantification of the data in (D). n = 3. *F*, HE staining of the brain after the first injection for 190 h. The scale bar represents 200 μm. *G*, fluorescence signal of P-H3 in the sixth-120 h brain (120 h after first injection for *dsGfp* control, and 190 h after first injection for *dsDelta* injection). The scale bar represents 200 μm. Fluorescence was detected using an Olympus BX51 fluorescence microscope (Olympus Optical Co.). *Red*, P-Histone H3 (P-H3) detected using anti-P-Histone H3 antibodies and goat anti-rabbit IgG secondary antibody labeled with DyLight594; *blue*, nuclei stained using DAPI. A: anterior, D: dorsal, L: lateral, P: posterior, V: ventral. *H*, fluorescence intensity statistics of G, n = 3. *I*, EdU staining DNA replication in the sixth-120 h brain (120 h after the first injection for *dsGfp* control, and 190 h after the first injection for *dsDelta* injection). Fluorescence was detected using an Olympus SpinSR10 laser confocal microscope (Olympus Optical Co). *Red*, EdU staining cells; *blue*, nuclei stained using DAPI. A: anterior, D: dorsal, L: lateral, P: posterior, V: ventral. The scale bar represents 200 μm. *J*, fluorescence points statistics of *I*, n = 3. *K*, qRT-PCR shows the expression of proliferation genes, n = 9 (three technical replications and three biological replicates). *L*, western blot and statistical analysis of the expression of P-H3. Samples were collected from the brains of sixth-120 h larvae in *dsGfp* and *dsDelta* by controlling sampling at the same developmental time (120 h after the first injection for *dsGfp* control, and 190 h after the first injection for *dsDelta* injection) in *K* and *L*. The statistical analysis was performed using Student’s *t* test (∗, *p* < 0.05, ∗∗, *p* < 0.01, ∗∗∗, *p* < 0.001, n = 3). The error bar indicates the mean ± S.D. *M*, a diagram showing the mechanisms by which the steroid hormone 20E promotes imaginal/adult neural cell proliferation. 20E *via* EcRA promoted the expression of SOX12, thus promoting the expression of the *Delta* during metamorphosis to form the imaginal/adult brain. DAPI, 4,6-diamidino-2-phenylindole; IgG, immunoglobulin G; qRT-PCR, quantitative real-time polymerase chain reaction.

To investigate the role of DELTA in adult brain development, *Delta* dsRNA was injected into sixth-instar 6-hour-old larvae to induce *Delta* knockdown. This manipulation resulted in significant larval mortality, with 34% of the treated larvae dying. In addition, 17% of the larvae exhibited a 20-h to 3 days delay in pupation (Fig. 8, *A*–*C*). *Delta* knockdown led to a marked reduction in brain size and severely impaired adult brain development (Fig. 8, *D* and *E*). HE staining revealed that the brains of *Delta*-knockdown larvae remained as the larval stage morphology, even developed to sixth-120 h post injection of dsRNA, whereas control brains had progressed to the adult brain morphology of P-2 d (Fig. 8*F*). Immunohistochemical analysis demonstrated a significant reduction in P-H3 signal in the brains of sixth-120-h postinjection larvae when the developmental time was the same (120 h after the initial *dsGfp* injection, and 190 h after the initial *dsDelta* injection) (Fig. 8, *G* and *H*). EdU staining analysis in the same samples revealed that DNA replication was repressed after the *Delta* knockdown compared to the control group (Fig. 8, *I* and *J*). Furthermore, the expression of pro-cell proliferation genes was downregulated (Fig. 8*K*), and cell proliferation, as indicated by P-H3 staining, was markedly suppressed in the *dsDelta*-injected group compared to the *dsGfp* control group (Fig. 8*L*). These findings collectively indicate that *Delta* plays a critical role in promoting cell proliferation in imaginal tissues, including the adult brain and midgut.

## Discussion

The underlying regulatory mechanisms governing animal brain postembryonic development remain poorly understood. Using the Lepidoptera insect, the cotton bollworm, as a model, this study demonstrates that SOX12 plays a crucial role in promoting neural cell proliferation during imaginal/adult brain development under steroid hormone 20E regulation. The 20E modulates *Sox12* expression *via* its nuclear receptor EcRA. In turn, SOX12 regulates *Delta* expression to facilitate neural cell proliferation in the developing adult brain during metamorphosis.

### SOX12 promotes adult neural cell proliferation

SOX family members are extensively studied transcription factors. In physiological conditions, they are involved in stem cell regulation during embryogenesis and adult tissue regeneration (21). In addition to their roles in embryonic stem cells, cell reprogramming, and development, SOX transcription factors have been demonstrated to be expressed in various stem and progenitor cell populations within adult tissues, where they play a crucial role in maintaining tissue-specific stem cells and ensuring proper progenitor differentiation (22). The functions of SoxNeuro and Dichaete during embryonic development and nervous system formation have been most extensively characterized in *Drosophila* (23, 24). SOX12 belongs to the SOXC superfamily and is involved in key functions in cell fate determination (25); however, little study reports its role in neural cell proliferation in post embryonic brain development. Our study demonstrated that SOX12 promotes imaginal neural cell proliferation during insect metamorphosis, revealing a key factor that promotes post embryonic adult brain development.

The larval nervous system of *Drosophila* is formed during embryogenesis and then grows in concert with the larval body until metamorphosis (9). The nervous system undergoes great changes during insect metamorphic development. The central nervous system (CNS) stores cells for building its adult circuits as the larva grows, and the nervous system does not discard all its larval neurons (26). The larval neurons are rapidly pruned after pupariation, followed by adult outgrowth, target recognition, and synaptogenesis. Indeed, most larval neural cells are preserved through metamorphosis and repurposed for adult use (23). Although larval neural cells are partially saved by reconstructing and reprogramming the function, the adult neural cells expand compared to the larval neural cells (7). The proliferation of neural cells derived from the progenitor cells of the imaginal brain constitutes a significant contributing factor. The majority of adult neurons are generated during larval and early pupal stages (24). The most difficult work is to distinguish the larval neural cells and the imaginal neural cells in the brain because they are chimeric in the brain. This study demonstrates that SOX12 promotes neural cell proliferation, and its proliferative-promoting characteristics render it a reliable biomarker of imaginal neural cells in the metamorphic brain.

The neural cell proliferation is also confirmed by EdU indication. EdU is a novel thymidine analog that is incorporated into DNA during the S phase of replication to label replicating cells and indicate DNA synthesis (27). The EdU staining indicates that this cell is in the process of replication, but it does not imply that it has undergone division. The phospho-histone H3 indicates chromosome condensation during mitosis and meiosis, indicating that the cell is entering the division process (28). EdU detected a strong signal in some regions, *e.g.*, the optic lobe region, which is likely due to cell proliferation plus endoreplication. Endoreplication is widespread in insects, plants, and mammals, leading to polyploidy as an advantageous strategy for the growth of terminally differentiated cells and tissues (29). Our results demonstrate that endoreplication also contributes to adult brain development.

The expression of SOX12 in other tissues, including the midgut and fat body, suggests it promotes imaginal cell proliferation in these tissues, too. Therefore, knockdown or knockout of *Sox12* also blocked cell proliferation in these tissues, which contributed to the phenotypes of pupation death or delayed pupation.

### 20E regulates the expression of *Sox12* through the nuclear receptor EcRA

The biological actions of 20E are through its cell membrane receptor, G protein–coupled receptor (GPCR) (30), and transmit the signal to its nuclear receptor (EcR) (31) to regulate gene transcription (32). Specifically, 20E binds to its nuclear receptor EcR, forming a transcriptional regulatory complex with ultraspiracle (USP), and subsequently binds to EcREs (33). In *Drosophila*, the EcR gene undergoes alternative splicing, generating three distinct isoforms—EcRA, EcRB1, and EcRB2—which differ primarily in their A/B domains (32, 34). EcRA, an isoform of EcRB, differs from EcRB primarily in its N-terminal region. Most insects possess two EcR isoforms, EcRA and EcRB, with EcRA predominantly expressed in adult tissues and EcRB highly enriched in apoptotic larval tissues (34, 35). Previous research has predominantly focused on the EcRB-mediated 20E signaling pathway, which promotes larval tissue programmed cell death by upregulating the expression of transcription factors such as *Br-C* and *Hr3* during metamorphosis (36, 37). EcRB1 is predominantly expressed in larval neurons immediately preceding metamorphosis (38) and plays a pivotal role in mediating neuronal pruning in the larval brain. Conversely, EcRA is primarily present in adult tissues and regulates neural cell proliferation in the brain during metamorphosis (9); however, the underlying mechanism is unclear. Our study reveals that 20E *via* EcRA upregulates *Sox12* expression and the downstream gene network, revealing the mechanism that 20E signaling through EcRA promotes imaginal neural cell proliferation.

### SOX12 upregulates the expression of *Delta*

Delta serves as the ligand for the Notch receptor, localized at the cell membrane, where it activates the Notch receptor in adjacent cells (trans-activation of Notch signaling). The Notch signaling pathway is widely recognized as a key regulator of NSC maintenance and neurodevelopment (39, 40). During CNS development, neurons arise from neural progenitor cells, and the Delta-Notch signaling cascade plays a pivotal role in governing cell fate decisions (41, 42, 43). Neuronal expression of Delta activates Notch signaling in neighboring tissues, thereby sustaining the proliferative state of adjacent cells (44). In *Drosophila*, lateral inhibition mediated by Delta and Notch regulates cell fate commitment not only in the CNS and peripheral nervous systems but also in diverse tissues, including the gut epithelium, musculature, malpighian tubules, and ovaries (43, 45). Similarly, in *Caenorhabditis elegans*, signaling through Delta- and Notch-related molecules governs a broad spectrum of cell-fate decisions across various tissues (46). Furthermore, Notch-Delta signaling functions at multiple developmental stages to orchestrate embryonic nervous system formation, including the selection of neural progenitor cells (neuroblasts; NBs), regulation of neuroblast daughter cell proliferation, neural cell fate specification, glial development, and axon pathfinding (47). Nevertheless, the transcriptional regulators governing *Delta* expression remain poorly characterized. In this study, we identified that SOX12 binds to the SOXBE element within the *Delta* promoter region and demonstrated that SOX12 directly enhances *Delta* transcription under 20E regulation. This finding represents a significant advancement in elucidating the upstream regulatory mechanisms controlling *Delta* transcription.

In addition, the transcriptome analysis further reveals a set of downstream genes regulated by SOX12, including *Raf*, *Dsor1*, *Ebi*, *Tbp*, *Delta*, and *Notch*. These genes decrease after *Sox12* knockout. RAF is a cytoplasmic serine/threonine protein kinase that plays essential roles in the transduction of a wide variety of growth-stimulating transmembrane signals (48). D-RAF is required during most of the developmental stages, functioning in the development of the compound eye, wing, and nervous system in *Drosophila* (49). Ras-activated DSOR1 promotes WNT signaling, which in turn promotes cell proliferation during *Drosophila* development (50). EBI is a nuclear-localized protein that plays an essential role in diverse signaling pathways and cellular processes. It is originally characterized as a positive regulator of the epidermal growth factor receptor (EGFR). EBI acts to activate (de-repress) transcription of Notch target genes important for *Drosophila* wing growth (51). TBP is one of the general transcription factors, which acts as a crucial mitotic bookmark for preserving the fate memory of *Drosophila* NSCs (52). *Delta* and *Notch* encode cell surface proteins that function in a wide variety of cell fate specification events during oogenesis, embryogenesis, and metamorphosis in *D. melanogaster*. Delta and Notch function as signal and receptor in cellular interactions required for the partitioning of fates among cells within equivalence groups in many developmental contexts (53). These data further demonstrate the network of neural cell proliferation promoted by SOX12 under 20E regulation.

## Conclusions

This study demonstrates that 20E upregulates the transcription of *Sox12 via* its nuclear receptor EcRA, which subsequently enhances the expression of the downstream proliferative genes, including *Delta*, thereby promoting neural cell proliferation in the imaginal/adult brain during metamorphosis, presenting a steroid hormone-EcRA-SOX12-DELTA-neural cell proliferation signaling axis in postembryonic brain development. Via this axis, 20E actively promotes adult neural cell proliferation for the adult brain development (Fig. 8*M*).

## Experimental Procedures

### Experimental animals

*H. armigera* were purchased from Keyun Biology on Taobao online and reared in our laboratory at 27 ± 1 °C with 60 to 70% humidity under a 14 h light and 10 h in the dark, and fed with artificial food according to a previously published method (54).

### HaEpi cell culture

The HaEpi cell line was established from the epidermis of fifth instar larvae in our laboratory (14). The cells were cultured in cell culture flasks at 27 °C with Grace’s medium containing 10% fetal bovine serum (Gibco). The cells were observed using an Olympus BX51 (Olympus Optical Co) fluorescence microscope.

### Quantitative real-time reverse transcription PCR

Total RNA was extracted by TRIzol reagent (TransGen Biotech), and first-strand complementary DNA (cDNA) was synthesized using 5 × All-In-One RT Master Mix (Abm) according to the manufacturer’s instructions. qRT-PCR was carried out using the qTOWER3/G system (Analytik Jena AG) with TransStart Tip Green qPCR Supermix (AiDLab). The relative expression levels of the genes were quantified using *Actb* (*β-actin*) expression as the internal control. Each experiment was repeated three times, with three parallel experiments each time. The 2^−ΔΔCT^ method was used to analyze the data (55).

### Preparation of rabbit polyclonal antibody against SOX12

A fragment of *Sox12* (nucleotide sequence 1 bp to 510 bp, amino acids 1–160), the molecular weight of SOX12 is 18 kDa, was inserted into plasmid pET-32a. The recombinant plasmid was sequenced and expressed in *Escherichia coli* strain Rosetta (DE3). Recombinant SOX12 protein was expressed in the supernatant. SOX12 was purified using a smart Ni-NTA beads 6FF column (SA005500, Smart-Lifesciences). Subsequently, 1 mg/ml protein was mixed with complete adjuvant in a 1:1 volume ratio and injected subcutaneously into New Zealand white rabbits. After 21 days, the renatured protein was mixed with incomplete adjuvant and injected subcutaneously. Seven days later, the renatured protein was mixed with incomplete adjuvant and administered intramuscularly. Seven days later, the blood was taken, and the supernatant was centrifuged to obtain anti-SOX12 antibodies.

### Western blot

Total protein was extracted from tissue or cells using 40 mM Tris–HCl or radioimmunoprecipitation assay (RIPA) lysis buffer, which contains 1 mM protease inhibitor PMSF to inhibit protease activity. After completely grinding the tissue, the homogenate was centrifuged at 10,000*g* at 4 °C for 10 min. The total proteins were measured by the BCA Method Micro Protein Concentration Determination Kit (Sangon Biotech) for quantification. Next, 30 μg of the protein sample was subjected to 7.5 to 15% sodium dodecyl-sulfate PAGE (SDS-PAGE) and transferred to nitrocellulose membranes. The membranes were incubated in blocking buffer (5% fat-free powdered milk in TBST (10 mM Tris–HCl, 150 mM NaCl, 0.02% Tween, pH 7.5) for 1 h at room temperature. The primary antibodies were diluted with blocking buffer and incubated with the membranes overnight at 4 °C. The membrane was washed with TBST twice and incubated with secondary antibodies (goat anti-rabbit/-mouse IgG horseradish peroxidase (HRP) conjugate secondary antibodies) diluted 1:5000. The membrane was immersed in High-sig enhanced chemiluminescence Western Blotting Substrate (Tanon Science & Technology), and the bands were detected by the enhanced chemiluminescence method.

### Immunohistochemistry

The tissue was placed in PBS and dissected carefully under a microscope. The tissue samples were immersed in 4% paraformaldehyde at 4 °C overnight. Samples were sent to Servicebio for hematoxylin and eosin (HE) staining. The tissues were cut into 4 μm sections using a paraffin-slicing machine. The sections were flattened in a 50 °C water bath and immediately adhered to a glass slide. The slides were dried at 70 °C overnight and subsequently dewaxed with Dewaxing transparent agent, Gradient Alcohol (100%-90%-80%-70%), and 1 × phosphate-buffered saline (PBS) (140 mM NaCl, 2.7 mM KCl, 10 mM Na_2_HPO_4_, and 1.8 mM KH_2_PO_4_). Then the slides were boiled in Sodium Citrate Antigen Retrieval Solution (Solarbio) for 20 min for antigen retrieval. The slides were incubated in blocking buffer (5% bovine serum albumin in PBS) for 30 min. The preserum and rabbit anti-SOX12 antibodies were diluted with blocking buffer (1:50) and incubated with the slides overnight at 4 °C, respectively. The slides were washed with PBS three times for 5 min each and incubated with fluorescent secondary antibody Alexa fluor 594-conjugated goat anti-rabbit IgG (H + L) (Biodragon Immunotech) diluted 1:500 for 1 h at room temperature. After being well-washed three times in PBS for 5 min each time, the slides were then stained with 4,6-diamidino-2-phenylindole (DAPI) (BBI LIFE SCIENCE) for 1 h at room temperature in the dark. An appropriate amount of blocking agent was added for blocking, and the samples were temporarily stored in a refrigerator at 4 °C to observe the fluorescence signal. The images were observed using an Olympus BX51fluorescence microscope (Olympus Optical Co).

### Whole-mount immunohistochemistry

The brain was fixed with 4% paraformaldehyde for 1 h at room temperature, followed by washing three times with PBS containing 0.5% Triton X-100 in PBS (PBSX) for 10 min each time. The brain was immersed in B-PBSX (PBSX containing 5% bovine serum albumin), gently shaken at 4 °C for 1.5 h, and the primary rabbit polyclonal antibodies anti-SOX12 were diluted in B-PBSX at a ratio of 1:50, or the rabbit anti-P-H3 was diluted in B-PBSX at a ratio of 1:200, and gently shaken at 4 °C overnight. The sample was washed three times with PBSX for 10 min each time. Fluorescently labeled secondary antibodies Alexa fluor 594-conjugated goat anti-rabbit IgG (H + L) diluted in B-PBSX at (1:500) were added away from light and gently shaken at room temperature for 3 h, washed three with PBS for 15 min each time, and DAPI to restain the nucleus at room temperature for 1 h, washed three with PBS for 15 min each time. An appropriate amount of blocking agent was added for blocking, and the samples were temporarily stored in a refrigerator at 4 °C to observe the fluorescence signal. The images were observed using an Olympus BX51fluorescence microscope (Olympus Optical Co).

### EdU incorporation

The brain was dissected in 50% Grace’s medium diluted in 0.1 M PBS (pH 7.4) as quickly as possible. Incubated the brain in a solution of EdU diluted in Grace’s medium (EdU working concentration: 20 μM) overnight at room temperature in a 96-well plate. The brain was washed 3 times with PBS for 10 min each time. Fixed the brain in a solution of 4% paraformaldehyde for 1 h at room temperature. The brain was washed three times with PBS containing PBSX for 10 min each time. Subsequently, 1% Triton X-100 was added in PBS (PBSX) and incubated at room temperature for 2 h. The brain was washed three times with PBS containing PBSX for 10 min each time and 1X Click-iT reaction cocktail (BeyoClick EdU Cell Proliferation Kit with AF594, Beyotime Biotechnology) was added and incubated for 30 min away from light at room temperature, washed three times with PBS containing PBSX for 10 min each time. DAPI was used to restain the nucleus at room temperature for 1 h. The images were observed using an Olympus SpinSR10 laser confocal microscope (Olympus Optical Co).

### RNAi in larvae

Primers with a T7 promoter were used to amplify the DNA templates of a complementary DNA by RT-PCR (Table S1). For the dsRNA transcription system, 2 μg of DNA template, 20 μl of 5 × transcription buffer, 3 μl of T7RNA polymerase (20 U/μl), 2.4 μl of A/U/C/GTP (10 mM) each, 3 μl of RNase inhibitor (40 U/μl, Thermo Fisher Scientific), and RNase-free water were mixed to a volume of 50 μl. After incubation at 37 °C for 12 to 14 h, 10 μl of RNase-free DNase I (1 U/μl, Thermo Fisher Scientific), 10 μl of DNase I buffer, and 30 μl of RNase-free water were added to the solution, which was incubated at 37 °C for 1 h. The product was purified with phenol/chloroform and precipitated with ethanol; the precipitate was resuspended in 50 μl of RNase-free water. The purity and integrity of the dsRNA were determined using agarose gel electrophoresis. A MicroSpectrophotometer (GeneQuant; Amersham Biosciences) was used to quantify the dsRNAs. The dsRNA was diluted to a concentration of 400 ng/μl with aseptic PBS. Five microliters of dsRNA was injected into the sixth instar larva at 6 h. Twenty-four hours after the first injection, 5 μl of dsRNA was injected for the second time, and repeated for a total of three injections. The same amount of *dsGfp* was injected as a control. Total RNA or protein was extracted from sixth-96 h of *dsGfp*, and the efficiency of RNAi was measured. The brains used for morphology were obtained 24 h, 72 h, 120 h, and 190 h after the first injection. HE staining was obtained from P-2 d of *dsGfp*, sixth-120 h of dsRNA samples. The P-H3 immunofluorescence staining was obtained from sixth-120 h of *dsGfp* and dsRNAs (120 h after the first injection for *dsGfp* control, and 190 h after the first injection for dsRNAs injection). Samples for the western blot of the expression of P-H3 were obtained from sixth-120 h of *dsGfp* and dsRNAs (120 h after the first injection for *dsGfp* control, and 190 h after the first injection for dsRNAs injection).

### CRISPR/Cas9-mediated knockout of *Sox12*

According to the N20NGG rule, seven sgRNA target sites, which were consistent with the sequencing results were designed using the Cas Designer website (http://www.rgenome.net/cas-designer/). The target sites that can be cut were selected based on *in vitro* digestion results. A 23 bp sgRNA sequence (target 5) in the second exon region of the *Sox12* gene was selected as the target of editing, which met the requirements of 5′-N20NGG-3′. The forward primer contained the T7 RNA polymerase recognition sequence (TTAATACGACTCA-CTATAGGG), 23 bp sgRNA sequence (ACAACCCAAACCACATAAAGAGG), and the 5′ sgRNA scaffold sequence (GTTTTAGAGCTAGAAATA), and the universal reverse primers of the scaffold sequence (AGCACCGACTCGGTGCCAC-TT) were used to amplify the sgRNA template (T7+sgRNA+scaffold), with the topo plasmid that containing the sgRNA scaffold as a template. The PCR product was used as a template for sgRNA synthesis *via* the MEGAscript T7 kit with T7 RNA polymerase (AM1333, Thermo Fisher Scientific). The fertilized eggs were collected within 2 h and arranged on glass slides for later microinjection. Cas-9 protein (200 ng/μl) and sgRNA (500 ng/μl) were thoroughly mixed, and 800 pL were injected into the newly fertilized egg. The phenotype was observed. For the WT group, five individuals were randomly selected for PCR product sequencing analysis. For the experimental group, 118 individuals with the phenotypes of pupation delayed and pupation death were all subjected to sequencing analysis of PCR products. The editing efficiency in population was calculated by the number of delayed pupation and pupation death divided by the number of sixth instar larvae × 100%. The region spanning the target sequence was amplified using PCR for sequencing. To evaluate the editing efficiency in an individual, the purified PCR products from one individual that had an abnormal phenotype were ligated to the topo plasmid and transformed into *E. coli*. Then, five colonies from one individual were randomly picked for sequencing to identify the mutations and nonmutations in an individual. A total of 40 individuals were used for individual editing efficiency analysis. The calculation method is the number of detected mutant colonies in an individual divided by the five colonies × 100 %±SD. Total RNA was extracted for qRT-PCR detection of the expression of *Sox12*.

### Hormonal regulation of SOX12

20E was stored at a concentration of 20 mM in DMSO. The 20E was diluted with PBS to a concentration of 100 ng/μl. Different doses of 20E, 100, 200, 300, and 500 ng were injected into the sixth instar 6 h larvae and were harvested after 6 h for dose analysis. Five hundred ng of 20E was injected into the larva and harvested at 3, 6, 12, or 24 h for time analysis. The equivalent diluted DMSO was injected as a control. The total mRNA was extracted for qRT-PCR to detect the mRNA level of *Sox12*.

### Overexpression of SOX12 in HaEpi cells

The ORF of *Sox12* was amplified using overexpression primers by RT-PCR (Table S1), and the RT-PCR product was inserted into the pIEx-4-GFP-His vector fused with GFP and His-tag. All recombinant plasmids in the experiments contained a His tag. Then, 5 μg of recombinant plasmids were transfected into HaEpi cells using the Quick Shuttle-enhanced transfection reagent (Biodragon Immunotech).

Total cellular proteins were extracted for western blotting or other experiments 72 h after transfection.

### CCK-8 detected cell viability

CCK-8 cell viability assay kit (C6005M, US Everbright Inc) contains WST-8 (2 - (2 - methoxy - 4 - (phenyl) - 3 - (4 - (phenyl) - 5 - (2, 4 - sulpho benzene) – 2 H - tetrazolium monosodium salt), which is reduced by dehydrogenase in the mitochondria to a highly water-soluble orange-yellow formazan product in the presence of electron carrier. The depth of the dye is proportional to the number of living cells. After adjusting the cell density to 4 × 10^4^ cells/ml, a 100 μl cell suspension was put into a 96-well plate, followed by 10 μl CCK-8, and incubated for 2 to 3 h in an incubator. When the color steadied, the absorbance value at 450 nm was determined by the Tecan microplate spectrophotometer (Tecan nanoquant M200pro, Hombrechtikon Männedorf).

### Luciferase activity assay

The promoter sequences of *Hr3*, *Sox12,* and *Delta* were predicted in the National Library of Medicine (NCBI), and the target fragments were amplified and sequenced, replacing the pIE promoter of pIEx-4 luciferase-GFP-His (LUCI-GFP-His) plasmid to construct *pHr3*-LUCI-GFP-His, *pSox12*-LUCI-GFP-His, and *pDelta*-LUCI-GFP-His reporter plasmids, respectively. In addition, the reporter plasmids for the deletion, mutation, and truncation 1, truncation 2, and truncation three of *pSox12*-LUCI-GFP-His were constructed, respectively. The reporter plasmids for the deletion, mutation, and truncation of *pDelta*-LUCI-GFP-His were constructed, respectively. Transcription factor-binding sites were predicted according to the JASPAR website (https://jaspar.genereg.net/). EcRB, EcRA, and SOX12 were inserted into pIEx-4-GFP-His plasmid, respectively, for overexpression. Then, pIEx-4-EcRB-RFP-His, pIEx-4-EcRA-RFP-His, and pIEx-4-SOX12-RFP-His plasmids were cotransfected with *pHr3-*, *pSox12*-, and *pDelta*-LUCI-GFP-His plasmids, respectively, paired with pIEx-4-Renilla luciferase thymidine kinase (pRL-TK) as an internal reference of the luciferase. After 24 h of cotransfection, cells were incubated with 20E (2 μM final concentration) or DMSO for 48 h. The expression of LUCI-GFP-His was detected by western blotting.

In luciferase activity assay, EcRB-RFP-His, EcRA-RFP-His and SOX12-RFP-His, pRL-TK, and *pHr3-*, *pSox12*-, *pDelta*-LUCI-GFP-His were cotransfected, respectively. After 24 h, the cells were incubated with 20E or DMSO for 24 h. Luciferase activity was measured using a Dual Luciferase Firefly and Renilla Assay Kit (UElandy) according to the instructions. After co-transfection for 48 h, the medium was removed, and cells were washed with PBS twice, then 1 × lysis buffer diluted with sterile water was added. The culture plate was shaken on a microshaker at room temperature for 1 h to fully lyse the cells. The lysate was collected, and the supernatant was transferred to a new Eppendorf tube. An EnSpire 2300 Multifunction Enzyme Reader (PerkinElmer) was used to measure the sample luciferase activity based on relative light units (RLUs) by adding 100 μl of 0.2 mg/ml firefly luciferase detection solution to each 100 μl sample. Then, 100 μl of 1 × coelenterazine working solution was added to each sample, and the RLU was measured. The RLU value of the firefly luciferase assay was divided by the RLU value of the Renilla luciferase assay. A comparison of the differences in the degree of activation of the reporter gene plasmids was based on ratios.

### ChIP assay

The EcRB-RFP-His, EcRA-RFP-His, and SOX12-RFP-His were constructed into pIEx-4-GFP-His and transfected into HaEpi cells, respectively, for 72 h. When cells were approximately 70% confluent, the cells were then treated with 2 μM 20E for 6 h to detect the binding of EcRB in the promoter of *Hr3*, EcRA in the promoter of *Sox12*, and Sox12 in the promoter of *Delta*. DMSO treatment was used as a solvent control. The ChIP Assay Kit (P2078, Beyotime Biotechnology) was used for the following assay according to the instructions. Anti-RFP antibody was used to coimmunoprecipitate EcRB-RFP-His, EcRA-RFP-His, SOX12-RFP-His, and their bound DNA, respectively. immunoglobulin G (IgG) was used as a negative control.

### RNA-seq data analysis

In addition, 10 ± 5 brains of P-2 d were collected for each group. The transcriptome sequencing was performed by Biomarker Genomics Institute (BGI) using the DNBSEQ T7 instrument. The *H. armigera* genome reference was downloaded from the website (https://www.ncbi.nlm.nih.gov/datasets/genome/GCF_002156985.1/), and the Gene IDs correspond to the National Center for Biotechnology Information (https://www.ncbi.nlm.nih.gov/). The raw data were filtered with SOAPnuke (https://github.com/BGI-flexlab/SOAPnuke) by (1) removing reads containing adapters (adapter contamination); (2) removing reads whose unknown base ('N' base) ratio is more than 1%; (3) removing reads whose low-quality base ratio (Base quality less than or equal to 15) is more than 40%, afterward clean reads were obtained and stored in FASTQ format. The clean data were mapped to the reference genome by HISAT (http://www.ccb.jhu.edu/software/hisat). The clean data were mapped to the assembled unique gene by Bowtie2 (http://bowtie-bio.sourceforge.net/Bowtie2/index.shtml). The FPKM values of genes and transcripts were calculated by software RNA sequencing (RNA-Seq) by Expectation-Maximization RSEM (http://deweylab.biostat.wisc.edu/rsem/rsem-calculate-expression.html). Using public database (KEGG, Gene Ontology [GO], and so on.) to annotate the genes. Between-group differential gene analysis was performed using the DESeq2 (http://www.bioconductor.org/packages/release/bioc/html/DESeq2.html) under the conditions of fold change ≥ 1 and adjusted Q-value ≤ 0.05. Poisson distribution was performed between-sample differential gene analysis under the conditions of fold change ≥ 1 and false discovery rate (FDR) ≤ 0.05. To gain insight into the change of genes, KEGG (https://www.kegg.jp/) enrichment analysis of annotated different expression gene was performed by Phyper based on the hypergeometric test in R software, and the *p*-value was calculated. Then, the *p*-value was corrected by false discovery rate to obtain the Q-value. The significant levels of terms and pathways were corrected by Q value with a rigorous threshold (Q-value ≤ 0.05). The heatmap of differential expression of genes was drawn according to the log_2_ (FPKM+1) and standardized to Z-scores across samples for each gene in different pathways by R software (https://posit.co).

### The commercial antibodies used in this study

The following antibodies were used in our study: rabbit anti Histone H3 (1:3000, 1768-1-AP, RRID: AB_2716755, Proteintech); rabbit anti Phospho-Histone H3 (Ser 10) (1:1000 for western blot and 1:200 for whole-mount immunohistochemistry, 9710S, Cell Signaling Technology); rabbit anti-actin (1:3000, AC026, RRID: AB_2768234, ABclonal Technology); rabbit anti-IgG (1:3000 for western blot and 1:200 for ChIP, AS061, ABclonal Technology); mouse anti GFP (1:3000 for western blot ABclonal Technology); mouse anti-RFP (1:3000 for western blot and 1:200 for ChIP, AE020, RRID: AB_2770409). Goat anti-rabbit IgG HRP conjugate secondary antibodies, diluted at 1:5000 (IH-0011, Dingguo Changsheng). Goat anti-mouse IgG HRP conjugate secondary antibodies, diluted at 1:5000 (IH-0031, Dingguo Changsheng). Alexa Fluor 594-Goat Anti-Rabbit IgG (H + L) (BD9279, Biodragon Immunotech).

### Statistical analysis

Excel and GraphPad 9 software (https://www.graphpad.com) were used for data analysis and graphing. All the experiments were repeated three times. qRT-PCR met the requirements of three biological and three technical replications. Significant differences between the two samples were tested using a two-tailed Student’s *t* test. ∗, *p* < 0.05, ∗∗, *p* < 0.01, and ∗∗∗, *p* < 0.001 present significant differences. In the figures, the bars represent the mean ± standard deviation (SD) for three independent biological replicates. The comparison between multiple sets of data was performed by analysis of two-way variance (ANOVA) with a Bonferroni-corrected *post hoc* test. The different lowercase letters showed significant differences (*p* < 0.05).

## Ethics statement

The antibody preparation in rabbits was in accordance with protocols approved by the Animal Care and Welfare Committee, Shandong University School of Life Sciences (SYDWLL-2021 54).

## Data availability

All data are contained within the article. The raw RNA-seq data generated from this study have been deposited in the Sequence Read Archive with the accession number PRJNA1498007. This article contains supporting information.

## Supporting information

This article contains supporting information.

## Conflict of interest

The authors declare that they have no conflicts of interest regarding the contents of this article.
