# Supplementary material for: SOX12 and DELTA mediate ecdysone-driven adult neural cell proliferation during metamorphosis
Source: J Biol Chem. 2026 Aug 7;302(9):113409. doi: 10.1016/j.jbc.2026.113409 (PMC13553576; doi:10.1016/j.jbc.2026.113409)
Supplement: Supplementary file 1 [file mmc1.docx]

**SOX12 and DELTA mediate ecdysone-driven adult neural cell proliferation during metamorphosis**

Lin Wang^1^, Li-Qiang Liang^1^, Pei-Yao Feng^1^, Yan Li^1^, Jin-Xing Wang^1^, Xiao-Fan Zhao^[1]^*

^1^ Shandong Provincial Key Laboratory of Development and Regeneration, School of Life Sciences, Shandong University, China

**Running title:** Cell proliferation in insect imaginal brain

**Supplemental Files**

**Supplemental Figures and legends: Figures S1-S15**

**Supplemental Table:**

**Table S1. List of primer sequences (Word)**

**Table S2. The data for Figure S2 (Excel)**

**Table S3. All genes of RNA-seq data (Excel)**

**Table S4. DEGs of RNA-seq data (Excel)**

**Table S5. Genes of KEGG pathway annotation classification (Excel)**

**Table S6. Genes of KEGG enrichment bubble chart (Excel)**

**Table S7. Genes of MAPK pathway (Excel)**

**Table S8. All genes of Spinocerebellar ataxia pathway (Excel)**

**Table S9. Genes screened by the Spinocerebellar ataxia pathway (Excel)**

**Table S10. The data for Figure S15 (Excel)**

**Figure S1. Phylogenetic analysis of SOXs.** MAGE 5 software was used to analyze the phylogenetic tree of SOXs in *H. armigera* and other species. The red triangle indicates SOX12 in this study.

**Figure S2. qRT-PCR screens for SOX highly expressed in the brain at the 6th-96 h metamorphic stage.** The heatmap was produced by the R programming language method based on the data of qRT-PCR (Table S2). The average values obtained from three independent qRT-PCR experiments, each of which used total RNA extracted from three different biological samples for the analysis. The number in the color bar indicates the expression levels.

**Figure S3. Alignment of SOXs.** The red boxed region indicates the antibody fragments. The yellow boxed region indicates the interference segments.

**Fig S4.** **Specific detection of antibodies.** A, specific detection of antibodies against SOX12, ACTB, P-H3, and H3. The 6th-72 h brain was used as the test sample. B, western blot detected SOX12 in HaEpi cells overexpressing SOX12-GFP using anti-SOX12 and anti-GFP antibodies. C, western blot detected SOX12 in HaEpi cells overexpressing SOX12-RFP using anti-SOX12 and anti-RFP antibodies. Secondary antibodies (goat anti-rabbit/mouse IgG HRP conjugate secondary antibodies).

**Fig S5. The location of SOX12 in the midgut was detected with immunohistochemistry.** HE staining showing the morphology of the midgut. Fluorescence was detected using an Olympus BX51 fluorescence microscope (Olympus Optical Co.). Red, SOX12 detected using anti-SOX12 antibodies and Alexa Fluor 594-Goat Anti-Rabbit IgG; blue, nuclei stained using DAPI. The bars represent 50 μm. IM, imaginal midgut; LM, larval midgut.

**Fig S6. Efficiency of knockdown, off-target.** A, efficiency of the knockdown of *Sox12* by qRT-PCR analysis. B, off-target assay. The statistical analysis was performed using Student’s *t* test (**p* < 0.05, ***p*<0.01, ***, *p* < 0.001, n = 9 (three technical replications and three biological replicates)) for A and B.

**Fig S7. CRISPR/Cas9 editing targets and mutant forms.** A, the seven target sequences of CRISPR/Cas9. B, in vitro enzyme digestion results in a nucleic acid gel map. The red arrow indicates that the targets were successfully cleaved. C, chromatogram confirmed by Sanger sequence from wildtype and *Sox12* mutant larvae. D, other mutation patterns identified via cloning and Sanger sequencing (nucleic acid sequences and corresponding amino acid sequences). Purple represents insertion.

**Figure S8. RNA-seq data analysis.** A, efficiency of the knockout of *Sox12* by western blot analysis. The samples were taken after pupation for 24 h by developmental time control. The statistical analysis was performed using Student’s *t* test (**p* < 0.05, ***p*<0.01, ****p*<0.001, n = 3). B, gene expression distribution in wildtype and mutation according to FPKM values. FPKM ≥10 indicates highly expressed genes, 1 < FPKM < 10 indicates moderately expressed genes, and FPKM ≤ 1 indicates lowly expressed genes. C, KEGG Pathway annotation classification.

**Figure S9.** Heatmap of genes in the MAPK signaling pathway in the fly, selected by KEGG in the data of RNA-seq. The genes are listed in Table S7. The heatmap was arranged by log_2_ (FPKM + 1) and standardized to Z-scores across samples for each gene. Red and purple indicate relatively high and low expression, respectively, with the color scale limited to −2 to 2.

**Figure S10.** Heatmap of genes in the Spinocerebellar ataxia, selected by KEGG in the data of RNA-seq. The genes are listed in Table S8. The heatmap was arranged by log_2_ (FPKM + 1) and standardized to Z-scores across samples for each gene. Red and purple indicate relatively high and low expression, respectively, with the color scale limited to −2 to 2.

**Figure S11. Classification of the EcRB and EcRA of *H. armigera*.** MAGE 5 was used to analyze the phylogenetic tree of EcRs in *H. armigera* with *Drosophila melanogaster* and *Bombyx mori* using the MEGA 5.0 software. The red vertical line indicates EcRB in this study, while the blue vertical line indicates EcRA in this study.

**Fig S12. Alignment of *H. armigera* EcR.**

**Fig S13. 20E through EcRA upregulated *Sox12* expression.** A, the expression of *Ecrb* in the epidermis, midgut, fat body, and brain. B, the expression of *Ecra* in the epidermis, midgut, fat body, and brain. 5 F: 5th instar feeding larvae; 5 M: 5th instar molting larvae; 6th instar 6 h larvae to 6th instar 120 h larvae; P-2 d to P-8 d: pupal stage at day 2 to day 8; F: feeding stage; M: molting stage; MM: metamorphic molting stage; P: pupae. The bar represents the mean value derived from three independent qRT-PCR replicates, each performed using total RNA extracted from three distinct biological samples. The error bar indicates the mean ± S.D. C, efficiency of the knockdown of *Ecrb* by qRT-PCR analysis. D, efficiency of the knockdown of *Ecra* by qRT-PCR analysis. E, off-target detection after knockdown of *Ecrb* in the brain. F, off-target detection after knockdown of *Ecra* in the brain. G, the endogenous expression of *Ecrb* in epidermal cells. H, the endogenous expression of *Ecra* in epidermal cells. The bars indicate the mean ± SD. Statistical analyses were conducted using Student’s *t*-test (**p* < 0.05, ***p*<0.01, ****p*<0.001). n = 9 for all graphs (three technical replications and three biological replicates).

**Fig S14. Schematic diagram of the *pHr3/pSox12*-LUCI-GFP-His reporter plasmid.** Plasmid maps of *pHr3*-LUCI-GFP-His, *pSox12*-LUCI-GFP-His, EcRB-RFP-His, EcRA-RFP-His and pRL-TK. EcRE was from the promoter of *Hr3.*

**Fig S15. Screening of proliferating genes.** A, heat map analysis of proliferation genes in the brain for 6th-24 h, 6th-96 h, P-3 d, and P-8 d from the database of transcriptome of the brain in our laboratory (Table S10). B, qRT-PCR analyzing the expression of *cMyc*, *Wnt*, and *Delta* in the brain for 6th-24 h and 6th-96 h. The statistical analysis was performed using Student’s *t* test (***, *p* < 0.001). n = 9 for all graphs (three technical replications and three biological replicates). The error bar indicates the mean ± S.D.

**Supplemental Table 1**

**Table S1** List of primer sequences.

|  | **Primer name** | **Forward sequence** | **Reverse sequence** |
| --- | --- | --- | --- |
| **qRT-PCR** | *Sox2* | CCAATCCAGCACTTTACACGA | GGGCAACGGTTCCTCCTTAG |
|  | *Sox5* | GAAACTGGCAAGCCTCACATC | TTGACATCGCCTTCCACCTG |
|  | *Sox8* | GGATCAAGATCGCAATCGGC | TTCATGGGCCTCTTGACGTG |
|  | *Sox14* | ATGGCATCGGATAACCCTAAA | CTTCGTCTTCCTCCTCGGC |
|  | *Sox12* | GGATGTGAGGTCCCTGTCG | GTTCTTCTTTGTCTGAGTGGCA |
|  | *Sox15* | ACCACGAAGATGGGTCAGGC | TTTTACTCAAATCGGCGTTATG |
|  | *Sox21B* | CATTGTTCCCGCCATTGC | GTGCCTTACTGAAGATTACCTGC |
|  | *Sox-domain* | GTCATACTCCGCCGTGCC | GAGTAGAACGATGACACCACGG |
|  | *Ecrb* | ATGTCCATAGAGTCGCGTTTA | TCACACGCTCCGGTATCC |
|  | *Ecra* | ATGGACTTGAAGCACGAGGTT | AGTACCCGTTCGGCTGC |
|  | *c-Myc* | ATGAAGACTGGAGGATAATGGA | TGTTGTGGGTGTAGTGGTTG |
|  | *Wnt* | GTCACGGTATGTCAGGCTCG | GGCTCCAGGTCAGTATTAGGC |
|  | *Delta* | GGGTTCTACGGGGAGCGAT | GGAGCGGCAGATTGGTTCA |
|  | *Sox21* | AAGAGGCTGGGAGCGGAG | CGGTATTTGTAGTCGGGGTG |
|  | *β-Actin* | CCTGGTATTGCTGACCGTATGC | CTGTTGGAAGGTGGAGAGGGAA |
|  | *B2-like* | TGATTCTCATTGTCGCTGTC | GCTTGCCACGCATACC |
|  | *C1-like* | GCTGCTGGTAGTAGTGTCG | AGGCAGCACTCGTCCA |
|  | *G1-like* | CGCCAAGTTCGCTATT | CAGACGGTAAATCACTAATC |
|  | *A1-homolog* | TGTGCCCTGATGACGGAG | TGACGACGAACCTCTTGC |
|  | *Igf-2-like* | CTGCCACGCTTACAACAGTCC | TCAGCAATACCGTTTCCCACT |
| **Exp in *E. coli*** | SOX12 | TACTCAGGATCCATGGTGCCCCAACAAGT | TACTCAGAGCTCCTCCATTGGTACACTTGGCA |
| **RNAi** | *Sox12* | GCGTAATACGACTCACTATAGGAGCAGACTCCCGACAT | GCGTAATACGACTCACTATAGGGTCTACCTCGCAGTCC |
|  | *Delta* | GCGTAATACGACTCACTATAGGAGTGTCACCGCTACCCC | GCGTAATACGACTCACTATAGGTCAGTCTCATTTTCCCC |
|  | *Ecra* | GCGTAATACGACTCACTATAGGCCCTACAGTCCTAATG | GCGTAATACGACTCACTATAGGTTTCTCTTTCCGTTTCAT |
|  | *Ecrb* | GCGTAATACGACTCACTATAGGCGCTGGTATAACAACGGAGGA | GCGTAATACGACTCACTATAGGAAGCTGGAGACAACTCCTCACG |
| **Overexpression** | SOX12 | TACTCAGAGCTCATGGTGCCCCAACAAGT | TACTCACTGCAGAGACGACGTCAGATACGAC |
| **ChIP-**  **qRT-PCR** | *Sox12E* | ACCAGCGTTTTAATATAAAAT | GCAGGAAAAGGGTCCCCA |
|  | *EcRE* | AAAGGCGGCGACGTAATATC | GTGTCGTGTACAGATATAGCGG |
|  | *EcRAE* | GCCATCATCTGAGAAGGATGG | GTCAACTAGGGCGAAACTTAGC |
| **Luciferase** | *pSox12*-LUCI | TACTCACCCGGGGAATAATGGTCACCACACCC | TACTCAGAGCTCCACCATGGTTTTTGTTTATCCAAAAA |
|  | *pDelta*-LUCI | TACTCACCCGGGAAAATTATACCAGCGTTTTAATATAA | TACTCAGAGCTCCACCATGGTGATGTCCCCATGTAGTGCA |
| **Sox12 sgRNA** | *Sox12-*sgRNA-1 | TTAATACGACTCACTATAGGGACAACCCAAACCACATAAAGGTTTTAGAGCTAGAAATA | AGCACCGACTCGGTGCCACTT |
|  | *Sox12-*sgRNA-2 | TTAATACGACTCACTATAGGGGAGGCCGATGAACGCGTTCAGTTTTAGAGCTAGAAATA | AGCACCGACTCGGTGCCACTT |
|  | *Sox12-*sgRNA-3 | TTAATACGACTCACTATAGGGTCTGGTCGCAGATAGAGCGCGTTTTAGAGCTAGAAATA | AGCACCGACTCGGTGCCACTT |
|  | *Sox12-*sgRNA-4 | TTAATACGACTCACTATAGGGCGATGAACGCGTTCATGGTCGTTTTAGAGCTAGAAATA | AGCACCGACTCGGTGCCACTT |
|  | *Sox12-*sgRNA-5 | TTAATACGACTCACTATAGGGATAGAGCGCAGGAAAATATGGTTTTAGAGCTAGAAATA | AGCACCGACTCGGTGCCACTT |
|  | *Sox12-*sgRNA-6 | TTAATACGACTCACTATAGGGTAGAGCGCAGGAAAATATGTGTTTTAGAGCTAGAAATA | AGCACCGACTCGGTGCCACTT |
|  | *Sox12-*sgRNA-7 | TTAATACGACTCACTATAGGGGACTCCAGACATGCACAATGGTTTTAGAGCTAGAAATA | AGCACCGACTCGGTGCCACTT |

**The GenBank numbers corresponding to the genes in the table.** XM_021343022.3 *Sox2*; XM_021330012.3 *Sox5*; XM_021339369.3 *Sox8*; XM_021327214.3 *Sox14*; XM_021334729.3 *Sox12*; XM_021341789.3 *Sox15*; XM_049852386.2 *Sox21B*; XM_021342971.3 *Sox-domain*; XM_021325641.3 *Ecrb*; XM_049839218.2 *Ecra*; XM_021331013.1 *c-Myc*; XM_021327154.3 *Wnt*; XM_021328717.3 *Delta*; XM_049835729.2 *Sox21*; XM_021337727.3 *β-Actin*; XM_021335312.1 *B2-like*; XM_021333700.3 *C1-like*; XM_021325930.3 *G1-like*; XM_021331808.3 *A1-homolog*; XM_021328915.3 *Igf-2-like*.

1. * Corresponding author: Xiao-Fan Zhao, E-mail: xfzhao@sdu.edu.cn
